# Accurate classification and prediction of knee osteoarthritis based on Al-Biruni Earth Radius metaheuristic optimizer and LSTM classifier

**DOI:** 10.1038/s41598-026-46131-7

**Published:** 2026-04-21

**Authors:** Amal G. Diab, El-Sayed M. El-Kenawy, Nihal F. F. Areed, Hanan M. Amer, Mervat El-Seddek

**Affiliations:** 1 Department of Communications and Electronics Engineering, MISR Higher Institute for Engineering and Technology, Mansoura, 35511 Egypt; 2 Department of Electrical Engineering, College of Engineering, Qassim University, Buraydah, 52571 Saudi Arabia; 3 Department of Electronics and Communications Engineering, Faculty of Engineering, Mansoura University, Mansoura, 35511 Egypt; 4 Department of Communications and Electronics Engineering, Delta Higher Institute of Engineering and Technology, Mansoura, 35111 Egypt; 5 Department of Electronics and Communications Engineering, Horus University, New Damietta, 34517 Egypt; 6 Jadara Research Center, Jadara University, Irbid, Jordan

**Keywords:** Knee osteoarthritis (KOA), Al-Biruni earth radius (BER), Machine learning (ML), Multilayer perceptron (MLP), Long short-term memory (LSTM), Computational biology and bioinformatics, Mathematics and computing

## Abstract

Knee osteoarthritis (KOA) is one of the worst varieties of arthritis. If not treated right away, it might lead to knee replacement. For this reason, early KOA detection is crucial for optimal therapy. This work tested and improved deep learning (DL) algorithms for predicting and identifying KOA. The suggested approach was evaluated utilizing an available dataset that included preprocessing approaches such as scaling and normalization. The Google-BER-LSTM hybrid model was explicitly designed to improve classification accuracy. The proposed binary optimization approach and other comparable methods include the Al-Biruni Earth Radius (BER), Harris Hawks Optimizer (HHO), JAYA Optimization Algorithm (JAYA), Satin Bowerbird Optimizer (SBO), Gravitational Search Algorithm (GSA), Stochastic Fractal Search (SFS), Multi-Verse Optimization (MVO), Biogeography-Based Optimizer (BBO), Whale Optimization Algorithm (WOA), Particle Swarm Optimization (PSO), and Thyroid Stimulating Hormone (TSH). The statistical study utilized ANOVA and Wilcoxon signed-rank tests to evaluate the efficacy and relevance of the suggested procedure to the ten additional methods. Furthermore, various visual representations were produced to demonstrate the suggested algorithm’s efficacy and resilience. As a result, the Google-BER-LSTM algorithm outscored the other optimizers on the bulk of the unimodal benchmark functions. For categorization, two machine learning (ML) models were utilized: multilayer perceptron (MLP) and long short-term memory (LSTM) network. The LSTM model had the best precision (PPV) of 0.9386792, negative predictive value (NPV) of 0.970845481, F-Score of 0.945368171, accuracy of 0.958558), sensitivity of 0.95215311, specificity of 0.973023881, and time of 428.4418 s. Thus, LSTM acted as a fitness function, with binary Al-Biruni Earth Radius (bBER) being used to optimize it. Finally, utilizing the suggested approach, KOA classification accuracy reached 0.995260664.

## Introduction

Osteoarthritis (OA) is a chronic degenerative illness characterized by cartilage deterioration, eventually progressing to bone degeneration. It is a disorder with many contributing causes, therefore challenging to identify, diagnose, and cure^1^. One of the deadliest types of arthritis is KOA, which involves deterioration of the knee’s articular cartilage. According to the World Health Organization (WHO)^2^, OA affects 500 million individuals globally, with epidemiological studies indicating that one in every three women and one in every five males over the age of 50 will develop KOA-related symptoms. 8.9 million fractures are reported globally, which is one per three seconds, with the hip, spine, and wrist causing a majority of these cases^3^. Hip fractures have extremely unfavorable outcomes; 20–24% of those affected die within a year after the fracture. KOA affects 22% of persons around 60 and over worldwide, with the frequency increasing to 34% in postmenopausal women^4^. A 2024 multicenter research of 15 nations found that one in every three KOA patients develops to fragility fractures, with 15.7% happening on the tibial plateau^5^. Notably, 40% of individuals with knee osteoporosis are initially misdiagnosed with osteoarthritis, which delays therapy. Emerging AI diagnostic technologies currently detect knee osteoporosis with 92% accuracy using standard X-rays. Such an increase may indicate that this disease affects older and younger people. According to the survey, 2 million of more than 8 million Americans with KOA are below 45, and the majority are under 65^6^. This condition affects about 250 million persons worldwide^7^. Up to 25% of individuals with OA are unable to do routine activities, and 80% of them will have mobility restrictions. Currently, visual observations of the closing of joint and osteophyte gaps in X-ray images serve as the basis for diagnosing OA, as well as radiological observation and assessment of problem severity.

Obesity, age, gender, knee traumas, and lifestyle are all likely risk factors for KOA, as noted in a recent study^8^. The long-term condition can cause joint deformity and dysfunction^9^; therefore, diagnosing OA before irreversible changes occur is critical for earlier intervention, which could be accomplished by permitting the visualization of knee tissue and measuring changes over time. Although its evolution may be slow and silent, some people experience rapid illness progression and severity^10–12^. However, with existing methods, OA is typically detected at the early to intermediate phases of the illness process, when patients find it more challenging to take preventative measures and, if they do, frequently fail to do so. The present methodologies of prompt OA detection, which rely on demographic and clinical data and, in some cases, auxiliary radiography, fall short of giving a definite and sensitive diagnosis. Pre-symptomatic illness identification may be possible by assessing the degeneration and the evolution of the knee anatomy. Therefore, it is crucial to visualize and measure the disease-affected knee tissues and their changes as time progresses. Because of a lack of medical facilities and knowledge in remote locations, OA is detected at advanced stages, when it has already begun to impair movement, and recovery is difficult. KOA is often diagnosed using symptoms, arthroscopy, X-rays, and magnetic resonance imaging (MRI) since the earliest phases of OA are typically hidden.

Furthermore, the image shows a poor link between the level of pain and functioning and how severe OA is. As a result, a more effective diagnostic technique is required to diagnose OA at its earliest stages. OA-related biomarkers can be helpful in this setting.

Although numerous algorithms based on deep learning have been proposed for diagnosing Knee Osteoarthritis (KOA), the majority of extant studies use end-to-end CNN classifiers that do not include systematic feature optimization or classifier parameter adjustment. Furthermore, little emphasis has been paid to combining adaptive metaheuristic-based feature selection with sequential learning models to improve discrimination and generalization performance. As a result, there is still a need for a strong and statistically validated hybrid framework that can successfully modify deep representations while optimising classification dynamics for accurate KOA grading. The study offers a novel hybrid deep learning (DL) framework for accurately predicting and grading Knee Osteoarthritis (KOA) using X-ray images. The suggested approach combines deep feature extraction, binary metaheuristic-based feature selection, and optimized sequential classification into a single diagnostic workflow. Unlike traditional CNN-based techniques, which rely purely on end-to-end classification, the proposed framework improves discriminative capabilities via adaptive feature refinement and classifier optimization.

The main contributions of this work are summarized as follows:

1. The creation of a comprehensive hybrid deep learning system that combines deep feature extraction, feature selection, and classification into a single optimized architecture for KOA diagnosis.
2. Pre-trained convolutional neural networks, such as AlexNet, VGG19, and GoogleNet, are used as deep feature extractors to capture high-level radiographic patterns associated with KOA severity.
3. The use of a binary BER technique for feature selection allows for successful dimensionality reduction by identifying the most informative subset of deep features, which improves generalization and reduces redundancy.
4. LSTM classifier parameters are optimized using the bBER algorithm, resulting in a novel BER-LSTM hybrid model with improved classification robustness and predictive performance.
5. The suggested GoogleNet-BER-LSTM model was thoroughly statistically validated, including one-way ANOVA and Wilcoxon signed-rank tests, to ensure performance stability and statistical significance.
6. An extensive compared investigation against existing DL-based KOA prediction models demonstrates the proposed framework’s superiority and reliability.
7. The superiority of the suggested framework is demonstrated by comparing its performance with alternative approaches.

The rest of the article is organized as follows: Sect.  2 covers current studies attempts in KOA diagnosis, Sect.  3 discusses the procedures used for the proposed technique and the feature selector algorithms, Sect.  4 demonstrates the evaluation criteria, Sect.  5 demonstrates the research’s significant results, and Sect.  6 discusses the study’s conclusion.

## Related works

Artificial intelligence (AI) is an umbrella term for technologies that mimic human intellect to automate jobs accurately. Several approaches to achieving this objective include developing procedures with clear guidelines and directions or using more clever algorithms built through ML. ML is an aspect of AI that utilizes procedures to autonomously learn via data, with incremental optimization and accuracy enhancements made throughout the training procedure^13,14^. DL is an ML technique that does not need a tagged or organized dataset^14,15^. Artificial neural networks (ANN) can learn the key aspects of a model without human intervention^14^. AI and ML modeling are increasingly utilized in orthopedics to aid in KOA assessment, pre-TKA planning, prognostication of disease development, and estimate of therapy outcomes. Developments improve the technology in technology and more datasets, but they still require further validation. DL models classify images from X-rays based on visual characteristics^16^, allowing healthcare practitioners to correctly recognize pneumonia, breast cancer, and bone fractures. Even so, there are challenges to using DL for X-ray image recognition, such as the enormous quantity of training data required and the possibility of model biases; such methods have demonstrated promise in enhancing the precision and effectiveness of medical imaging analysis. One promising strategy for raising the precision of X-rays recognition is transfer learning (TL) based feature engineering^17^.

In image classification applications, TL involves extracting the best-fit features of picture data using pre-trained neural networks (NN)^18^. The final network layer’s outputs are utilized as a new classifier’s characteristics, and the weights and architecture of the pre-trained networks are frequently frozen. A neural network might employ a transfer approach to extract significant characteristics from X-rays^19^. TL feature engineering enables researchers to tailor pre-trained networks to specific objectives^20^. Thus, TL feature engineering has proved to encourage outcomes in raising X-ray identification accuracy and is expected to remain an essential technique in medical imaging research^21^. Recent research has used convolution neural networks (CNNs) to determine how severe OA is automatically. This study investigated the influence of multiview images, previous information on diagnosis accuracy, and the efficacy of a DL procedure according to plain radiographs in detecting KOA.

Metaheuristics are crucial to maximize the efficacy of KOA classification systems. Metaheuristic procedures are utilized to improve the efficacy of standard KOA categorization approaches by optimizing parameters, setups, and decision limits^22^. Metaheuristics are especially effective when dealing with complex or high-dimensional databases, where standard approaches may have limits. In these scenarios, metaheuristics offer a flexible and dynamic means of enhancing and optimizing classification algorithms^23^. Particle Swarm Optimization (PSO), Ant Colony Optimization (ACO), Simulated Annealing (SA), Harmony Search (HS) algorithm, and Grey Wolf Optimizer (GWO) are a few examples of metaheuristics designed for categorization. Metaheuristic approaches contribute sophistication through improved navigation of the solution space. This usually produces better resilience, processing speed, and classification accuracy. Incorporating metaheuristics in categorization problems demonstrates a planned and adaptable strategy for addressing problems. Metaheuristics enables ML approaches to handle the complexities of complicated data patterns with more adaptation and effectiveness by adopting various solutions and overcoming the limits imposed by standard techniques.

The binary BER^24^ method proved the most successful regarding the algorithms tested, demonstrating proficiency in parameter adjustment and feature selection (FS). BER’s demonstrated usefulness in various fields, covering engineering, healthcare, and finance, made it ideal for this study. The fundamental goal of combining ML with a metaheuristic procedure such as BER is to improve the precision of diagnosis. The collaboration enhances the model’s capacity to recognize intricate patterns, allowing for more accurate evaluations and timely treatments. Many studies shown below was described and compared to identify and classify KOA utilizing various ML, and DL models. Table 1 provides an overview significant research on the diagnosis of KOA.

In 2023^25^, Serafeim Moustakidis et al. deployed Dl and ML models to handle the diagnostic problem of KOA. Accuracy, confusion matrix, two fairness metrics-demographic parity (DP) and balanced equalized odds (BEO) were all included in the hybrid criterion. To illustrate the efficacy of the suggested technique, several subgroups of control subjects were selected using self-reported clinical data. The DNN with the highest performance approach was compared to various prominent and well-known ML classification algorithms regarding accuracy and fairness. The proposed technique achieved a categorization accuracy of 79.6% and fairness metrics of BEO: 92% and DP: 98.5%. However, the achieved accuracy remains relatively moderate compared to recent DL-based frameworks, and the study did not incorporate explicit deep feature optimization or metaheuristic-based tuning to enhance model generalization.

In 2023^26^, J. Hirvasniemi et al. structured the KOA prediction (KNOAP2020) challenge, which aimed to objectively assess techniques for forecasting the occurrence of symptomatic radiographic KOA within 78 months utilizing a blinded ground truth test set. Participants in the competition trained their models utilizing accessible data sources. All challenge participants received access to a test set of 423 knees, which included MRI and X-ray data. Images were transformed to NIfTI format, saved and shared using the Health-RI XNAT platform. They evaluated the efficiency of the presented frameworks utilizing the area under the receiver operating characteristic curve (ROC-AUC) and balanced accuracy (BACC)—the bulk of submissions employed DL to extract information from images. Except for UC-MRI, all frameworks were trained on the OAI dataset. Various approaches were used, including XG-Boost classifier, Logistic regression, MLP Gradient boosting machine, Res-Net18, Gaussian Naïve Bayesian (GNB), ensemble classifier, and linear discriminant analysis. Despite the extensive benchmarking effort, multiclass grading proved tough, with numerous submissions reporting moderate balanced accuracy values, underscoring the intrinsic difficulties of long-term KOA prediction and generalization across datasets.

In 2023^27^, Nacer Farajzadeh et al. introduced a deep residual NN called IJES-OA to assess the severity of KOA independently. The network was designed to focus on distinguishing the margins of the bones in the knee joint. Results from experiments using the MOST dataset for training and the OAI dataset for validation and testing showed that IJES-OA was less complex than other methods while achieving high average accuracy and precision scores of 80.23% and 0.802, respectively. Nonetheless, the stated accuracy shows that reliable multiclass severity grading is still a difficult process, especially when validating across many datasets.

In 2023^28^, Yun Xin Teoh et al. proposed a multitasking framework utilizing CNN feature extractors and ML classifiers to recognize 9 key characteristics of OA. They presented a novel FE approach by switching a connected layer with a global average pooling (GAP) layer. The effectiveness of three ML classifiers, Random Forest (RF), Logistic Regression, and K-Nearest Neighbor (KNN), and sixteen distinct CNN feature extractors from the VGG, Efficient-Net, Res-Net, and Dense-Net families which are trained on the ImageNet dataset was compared. The VGG16-GAP feature extractor and KNN classifier produced an optimal approach with a classification accuracy of 92.97%, demonstrating CNN feature extractors’ potential for multifunctional diagnosis. However, the framework was primarily based on traditional ML classifiers without adaptive optimization techniques, which may restrict feature refinement and resilience.

In 2023^29^, Abdul Haseeb et al. developed an innovative technique to forecast and classify KOA using DL and WOA optimizers. Two pre-trained DL frameworks, Efficientnet-b0 and Densenet201, were employed for FE. Fixed hyperparameter values in deep TL were utilized to train these picked frameworks on X-rays. In the following stage, fusion was conducted utilizing a canonical correlation technique, resulting in a feature vector with additional details beyond the initial. Following that, an enhanced WOA was created for dimensionality elimination. Finally, the selected features were fed into ML algorithms like Fine-Tuned support vector machine (SVM) and NN for categorization. The categorizing accuracy of the suggested procedure was 90.1%. Although WOA-based dimensionality reduction was used, the study did not include statistical significance testing or robustness evaluation across different data splits.

In 2023^30^, N. Hema Rajini and A. Anton Smith suggested a novel PSO approach alongside Deep Neural Network (DNN), known as the PSO-DNN approach, for identifying and categorizing KOA from X-rays in an IoHT environment. The X-rays were obtained in the DICOM format from individuals and converted to greyscale images for additional processing. Adaptive histogram equalization algorithms and a guided filter (GF) were employed to improve the images and remove noise. Curvature values were calculated and the regions of the synovial cavity were extracted from the picture using a segmentation procedure in mind global thresholding. The suggested approach obtained an accuracy of 89.54%. The PSO-DNN framework optimizes deep neural network parameters and achieves high classification accuracy (~ 89.54%). However, the approach requires extensive preprocessing, which may increase computational complexity and make generalization to different datasets challenging.

In 2023^31^, A. D. Goswami utilized the CNN Inception Net V2 approach to categorize and assess the degree of damage in knee X-rays. The first stage entailed segmenting the images to establish the exact position of the knee for the CNN model’s severity level. They then utilized contour detection to detect the edges of the knee’s target segmented area. In order to raise the quality of the images and give a decent FE method for the CNN Model, they adopted the image enhancement procedure, commonly known as image sharpening. Following that, the images were assigned a five-point severity rating. They obtained a mean accuracy of 91.03% on improved images. Although enhanced image sharpening improved CNN classification accuracy, the method relies on numerous preprocessing stages, was assessed on a single dataset, and lacks rigorous statistical validation, thereby limiting generalizability and reproducibility.

In 2023^32^, Liu J et al. assessed 4,200 paired knee joint X-rays utilizing the DL procedure to evaluate the effectiveness of anteroposterior and lateral plain radiographs in conjunction with previous zonal segmentation to identify KOA. DL algorithms were sorted into four categories according to if they utilized automatic zonal segmentation and multiview images as their DL prior knowledge. Four independent DL frameworks were assessed for diagnostic efficacy utilizing receiver operating curve analysis. With multiview images and prior information, the DL system’s overall accuracy was 0.96, whereas an experienced radiologist was 0.86. The study found that combining multiview images and zonal prior knowledge improved deep learning classification performance, but it only looked at a single dataset and did not explore external validation or generalization to other clinical settings.

In 2023^33^, J. Song and R. Zhang proposed a DL-based computer-aided KOA identification approach based on multivariate data. The deployed DL algorithms utilized data from physiological signals containing multivariate information. The research results indicated that, while the augmented DL technique accomplished a 93% performance score, it may still be improved to achieve its full potential. Although the model performed well using multivariate physiological signals, it was not evaluated on standard X‑ray images, limiting comparability with typical KOA imaging studies.

In 2023^34^, Amjad Rehman et al. proposed a CNN-Random Forest-K-neighbors (CRK), a revolutionary feature engineering strategy involving TL, accurately identifies OA by intelligently extracting spatial characteristics from X-rays utilizing a 2D-CNN. The spatial information was fed into the RF and k-neighbor’s procedures, which generated a probabilistic feature set. The probabilistic feature set was then used to create the deployed ML-based approaches. The hybrid TL-CNN with Random Forest and KNN demonstrated high performance, however the strategy adds increased model complexity and lacks comprehensive statistical or cross-dataset validation to ensure robustness and generalization.

In 2023^35^, Tariq T et al. presented a single posteroanterior standing knee x-ray foundation for an automatic DL-based ordinal categorization system for KOA early diagnosis. A collection of knee joint X-rays from the OAI was utilized for this analysis. They merged ResNet-34, VGG-19, Dense-Net 121, and Dense-Net 161 into an ensemble and utilized TL to enhance the model’s performance. This method yielded an overall accuracy of 98% and a 95% confidence interval for a quadratic weighted kappa of 0.99. Despite achieving 98% accuracy, the ensemble of CNNs is computationally demanding, hindering practical implementation.

In 2023^36^, Nasser Y et al. evolved the Discriminative Shape-Texture-CNN (DST-CNN) that detected KOA from X-rays. The suggested framework incorporated a discriminative loss to increase the separability of classes and address notable inter-class similarities. The CNN procedure incorporated a unique Gramme Matrix Descriptor (GMD) block. It computed texture information from several levels below and combined it with the highest layers’ form data. DST‑CNN improves early KOA diagnosis but struggles with very similar classes and requires large training data.

In 2023^37^, Chen N et al. proposed YOLO version 3 (YOLOv3)-based innovative modelling technique for the automated, simultaneous localization of knee joints and quantification of radiography KOA. YOLOv3 is a sophisticated deep CNN method for recognizing targets that, as its special residual connection and feature map merging, allows for simultaneous micro-object detection and quantification. Thus, utilizing the YOLOv3 architecture, a unified CNN technique was developed to integrate knee joint recognition and related Grading of the seriousness of OA. They graded KOA with a desirable accuracy utilizing public and clinical facts. Although the YOLOv3-based method offers fast automatic KOA grading, it concentrates on localization and single-step processing and may not provide extensive evaluation of severity discrimination performance across typical multiclass grading ranges.

In 2023^38^, Mohammed AS et al. recommended utilizing KOA images from the OAI dataset to detect the illness employing 6 pre-trained DNN frameworks: VGG16, VGG19, ResNet101, MobileNetV2, Inception-ResNetV2, and DenseNet121. They conducted two classifications: a binary classification, which decided if KOA was present, and a three-class classification, which indicated the severity of KOA. For a comparison investigation, they investigated three datasets, each having five, two, and three classes of KOA photographs. Their classification accuracy with the ResNet101 DNN model was 69%, 83%, and 89%, respectively. Although pretrained residual networks achieved up to ~ 89.9% accuracy for severity classification, the method depended largely on preprocessing and did not address dataset imbalance or provide thorough generalization evaluation.

In 2023^39^, Sajaan Almansour SH et al. presented a CNN approach to categorize KOA into five categories utilizing X-rays. Furthermore, two pre-trained TL approaches, Xception and Inception Res-Net V2, were compared to the proposed CNN model. These strategies were assessed regarding precision, recall, F1 score, and accuracy. With 98% accuracy, the proposed approach outperformed both TL algorithms. Although the custom CNN achieved high accuracy for KOA severity classification, it relies on a single model without extensive evaluation of generalization across diverse datasets.

In 2023^40^, Lee S. and Kim N. classified degenerative arthritis into Kellgren-Lawrence (KL) classes using DL techniques on X-ray images. They evaluated various models, including VGG-Net, Dense-Net, Res-Net, and others, using a standard dataset for osteoarthritis classification. Their weighted ensemble method achieved 72.8% accuracy on testing dataset, surpassing the existing state-of-the-art performance by about 1%. Although the ensemble of multiple deep models modestly improved classification performance, the overall accuracy remained moderate and the study did not explore detailed robustness evaluation across different datasets.

In 2024^41^, Isra Malik et al. presented a completely automated computer-aided diagnostic (CAD) approach for accurately assessing KOA seriousness. The suggested CAD approach utilized an ensemble TL technique to retrieve powerful deep features by combining several DL models. This approach integrated features from two AI approaches: (1) Alex-Net, which extracted implicit class-wise deep features by preprocessed data, and (2) a proprietary Isr-Net, which added further feature depth. Unsupervised k-means clustering with PCA dimensionality reduction divided each class into subgroups, further refining characteristics. The ACO optimizer determined highly instructive traits. The model was assessed utilizing the OAI dataset. It achieved average overall accuracies of 89.89% and 85.44% with SVM and KNN classifiers. Although the high classification accuracy that the system achieved, it requires numerous feature fusion and optimisation processes and lacks external validation for generalisation.

In 2024^42^, S. Kavitha et al. presented an optimized FS strategy to retrieve crucial data from X-rays. They developed a DL algorithm based on radiometric data to identify the KOA stage reliably. First, they analyzed the X-rays to remove noise. Second, they extracted characteristics from X-rays using texture and color-based approaches. Third, they employed the firefly methodology to choose the most associated characteristics. Finally, they used a CNN model to identify the KOA stage. The CNN was trained and validated using the outcomes of FE, both with and without FS. When applied to two separate inputs, the measures were used to verify CNN’s effectiveness. The experiment findings showed that the FS optimization strategy improved the accuracy of the CNN model by 2.5%. Although optimization techniques were applied to enhance KOA classification efficiency, the study lacks detailed performance evaluation and comparison with deep learning‑based severity grading approaches.

In 2024^43^, B. Subha et al. suggested a novel Gaussian Aquila Optimizer (GAO) relayed on Dual Convolutional NN (DCNN) for recognizing and categorizing OA X-rays. The GAO was designed to incorporate Gaussian mutation during the exploitation step of the Aquila optimizer, resulting in the best global optimum value. The weight and bias parameters of the new DCNN approach were optimized by the suggested GAO, which was meant to equalize the convolutional layers in every convolutional approach. The knee dataset consisted of 2283 images. Each image had a width and height of 512 × 512 pixels. The suggested innovative GAO-DCNN procedure achieved categorization scores for aberrant knee case-knee joint images with 98.77% categorization accuracy, 98.25% sensitivity, and 98.93% specificity. GAO‑DCNN achieved high accuracy but relies on complex ensemble optimization, limiting generalization.

In 2024^44^, Teemu A. T. et al. graded future KOA into three severity degrees utilizing the KL scale. Their two-stage technique, which splits the classification effort into two binary classifications was prompted by the difficulty of multiclass classification. Their ML approach utilized two balanced RF algorithms. The OAI provided a longitudinal 8-year analysis for 1213 knees as part of the training dataset. The knee joint dimensions were estimated using a self-coded MATLAB graphical user interface. Two balancing RF categorization approaches were trained on 500 trees utilized along with 10-fold stratified cross-validation. A balanced accuracy of 65.9% and a weighted F1 score of 79.0% were obtained from the categorized approach. The two-stage MRI model predicts KOA severity well but was evaluated on a single dataset, lacking external validation.

In 2025^45^, A. K. Mahapatra et al. introduced a Fast Flying Particle Swarm Optimisation (FF PSO) technique to improve traditional PSO for limited optimisation and feature selection challenges. FF PSO enhances convergence while balancing exploration and exploitation, resulting in competitive performance on benchmark optimisation tasks. However, it does not cover deep feature representations, medical imaging classification tasks, or robustness/generalization evaluations.

In 2025^46^, A. K. Mahapatra et al. presented a hybrid strategy for feature selection and neural network training called Quantized Orthogonal Experimentation SSA (QOX SSA), which combines the Salp Swarm Algorithm with orthogonal experimental design. While QOX SSA increases exploration-exploitation trade-offs and NN training efficiency on standard datasets, it does not assess deep feature representations, medical image classification, resilience, or cross-dataset generalization.

In 2025^47^, N. Panda et al. developed Adaptive Dimensional Search SSA (ADOX SSA), which combines orthogonal experimentation with adaptive dimensional search within SSA to improve feature selection and train RBF neural networks. Although this strategy improves solution quality and search efficiency on benchmark tasks, it is tested in non-medical settings and does not include measures of robustness, generalization, or uncertainty.

In 2025^48^, N. Panda et al. developed an improved Salp Swarm Algorithm (SSA)-driven deep CNN for brain tumor classification, improving CNN performance through SSA-based optimization. Despite producing great results on MRI tumor data, the study does not focus on osteoarthritis X-ray pictures, nor does it incorporate robustness, cross-dataset generalization, or uncertainty analysis.

In 2025^49^, N. Panda et al. introduced a Quantum-inspired Adaptive Mutation Operator-enabled PSO (QAMO PSO) for parallel optimization and parameter adjustment of Kolmogorov-Arnold networks. While QAMO PSO improves convergence and optimization efficiency, its priorities parameter tuning over feature selection or medical image classification, and it lacks robustness and cross-dataset evaluation.

In contrast, BER-LSTM combines deep feature extraction and sequential classification, as well as robustness, uncertainty, and generalization assessments, with a focus on osteoarthritis X-ray severity analysis.

**Table 1:** A summary of related literature.

Reference	Dataset (Name/Size)	Models	Purpose	Accuracy
Serafeim Moustakidis et al., 2023^25^	Subgroups of control participants from self-reported clinical data.	DNN ML models DP and BEO	Diagnose KOA	79.6%
J. Hirvasniemi et al., 2023^26^	OAI/423 MRI and X-ray images KNOAP/30	XG-Boost classifier Logistic regression MLP classifier Gradient boosting machine ResNet-18 GNB Gradient boosting machine Ensemble classifier Linear discriminant analysis	Diagnose KOA	
Nacer Farajzadeh et al.,2023^27^	MOST (training) OAI (validation and testing)	IJES-OA Net	To determine the KOA seriousness.	80.23%
Yun Xin Teoh et al., 2023^28^	OAI/ 9,592 knees	CNN VGG Efficient-Net Res-Net Dense-Net ML classifier: RF, LR and KNN	To identify nine crucial OA characteristics.	VGG16-GAP (KNN classifier): 92.97%
Abdul Haseeb et al., 2023^29^	X-Ray images	WOA optimizer Efficient net-b0 Densenet201 SVM	Diagnose KOA	90.1%
N. Hema Rajini and A. Anton Smith, 2023^30^	X-rays from patients	PSO optimizer GF Adaptive histogram equalization. Global thresholding	Diagnose KOA	89.54%
A. D. Goswami, 2023^31^	OAI	Inception Net V2 VGG-Net Res-Net Dense-Net Contour detection Image sharpening	Diagnose KOA	91.03%.
Liu J et al., 2023^32^	Fifth Affiliated Hospital of Sun Yat-sen University (Zhuhai, China)/4,200 X-ray images	Zonal segmentation U-Net ResNet-50 Grad-CAM	Diagnose KOA	97%
J. Song and R. Zhang, 2023^33^	Vibroarthrographic (VAG) database.	LD-S based strategy AMD-CNN	Diagnose and grade KOA.	Automatic detection accuracy = 93.6%. Early detection accuracy = 92.1%. Grading detection accuracy = 84.2%.
Amjad Rehman et al., 2023^34^	3615 images	2D-CNN CRK model CNN ML classifier: RF and KNN	To determine the seriousness of KOA at its infancy.	99%
Tariq T et al., 2023^35^	OAI/3857 images	Fine-tuned ResNet-34 VGG-19 Dense-Net 121 Dense-Net 161	Diagnose KOA	98%
Nasser Y et al., 2023^36^	OAI MOST	Discriminative Shape-Texture. DST-CNN	Diagnose KOA	
Chen N et al., 2023^37^	-	YOLOv3	Automatically localizing knee joints and measuring radiographic KOA at the same time.	
Mohammed AS et al.,2023^38^	OAI/9786 knee images	VGG16 VGG19 ResNet101 MobileNetV2 Inception ResNetV2 DenseNet121	Diagnose KOA	Resnet 101 Accuracy= 69%, 83%, and 89%,
Sajaan Almansour SH et al.,2023^39^	8381 X-ray image	Xception Inception Res-Net V2	Diagnose KOA	98%
Lee S and Kim N, 2023^40^	8260 X-ray images	VGG-Net Dense-Net Res-Net Tiny-Net Efficient-Net, Mobile-Net, Xception, and ViT	Assign KL scores to degenerate arthritis.	72.8%
Isra Malik et al., 2024^41^	OAI	Ensemble TL-ACO Alex-Net custom Isr-Net k-means clustering based on PCA ACO optimizer	Diagnose KOA	SVM: 89.89% KNN: 85.44%
S. Kavitha et al., 2024^42^	Kaggle X-rays	Texture- and color-based FE. CNN. Firefly approach optimization.	Diagnose KOA	CNN approach is improved by 2.5%
B. Subha et al., 2024^43^	Captured from the humans/2283 X-rays.	GAO Optimizer DCNN	Diagnose KOA	98.77%
Teemu A. T. et al., 2024^44^	OAI/1213	RF Self-coded MATLAB graphical user interface	Diagnose KOA	65.9%

## The proposed system

**Fig. 1:**
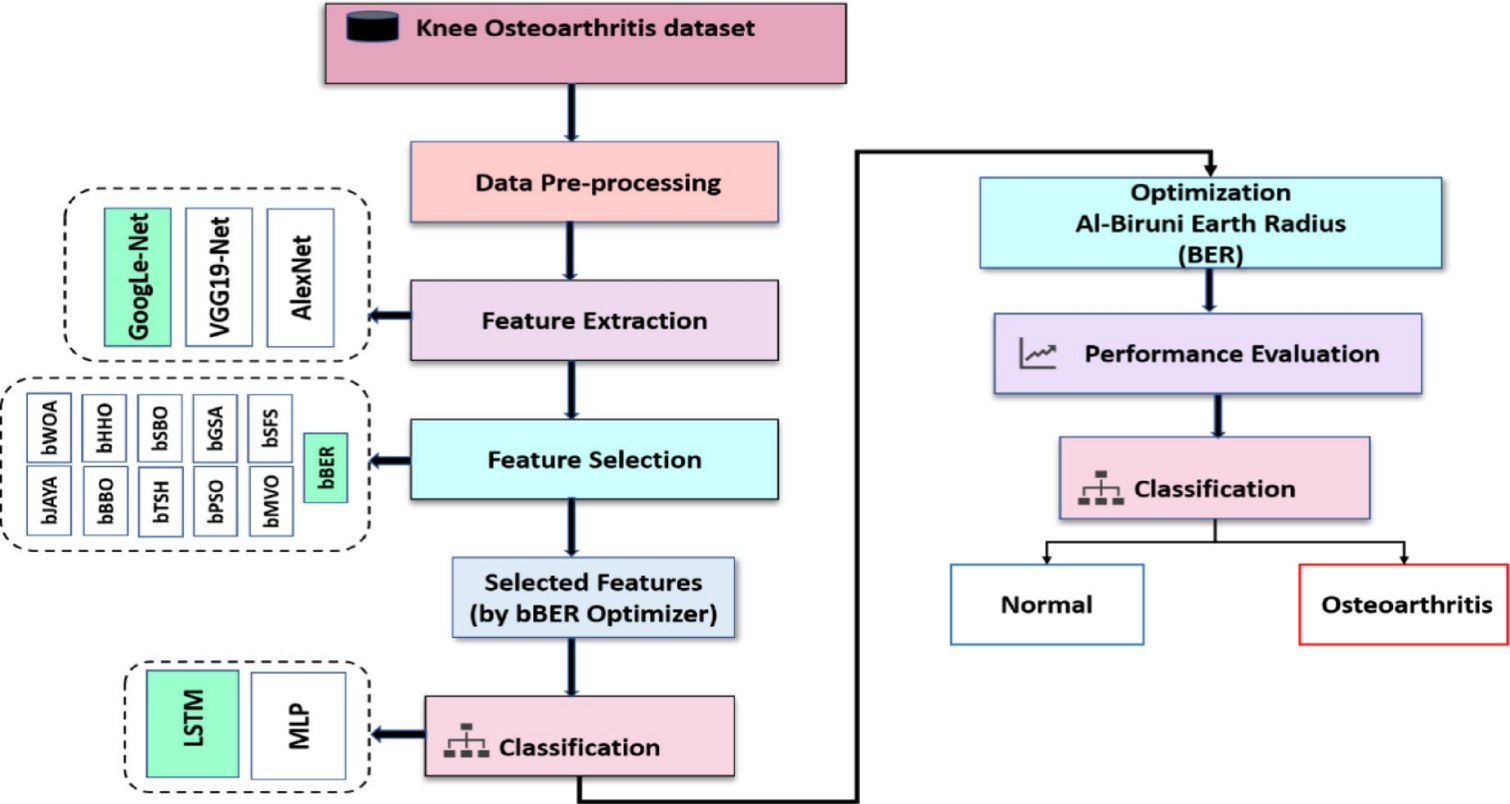
The proposed framework for classifying and detecting KOA.

Figure 1 shows an illustration of the suggested framework’s sequential procedure. The methodological structure of this study begins with a first step devoted to thorough data preparation, which includes scaling, normalization, and null entry removal. Followed by the FE step, which is performed using Alex-net, Google-net and VGG19. The implementation of FS approaches, which use eleven binary-form optimization strategies, is crucial to this phase: BER, HHO, JAYA, SBO, GSA, SFS, MVO, BBO, WOA, PSO, and TSH. The study employs the suggested FS technique in the next step, utilizing the binary encoding of BER to separate the most pertinent characteristics. This step is essential for determining the best features and improving classification accuracy by eliminating unnecessary or irrelevant data items. A suite of ML models is then used to classify the revised dataset, with the classifiers chosen based on the results of FS. This study’s ensemble of classifiers includes LSTM and MLP. The suggested technique is used to fine-tune LSTM hyperparameters to guarantee optimal performance. To rigorously quantify the uncertainty of the reported model performance metrics, 95% confidence intervals (CIs) were computed for Accuracy (ACC), Sensitivity (TPR), Specificity (TNR), Positive Predictive Value (PPV), Negative Predictive Value (NPV), and F1-score using the normal approximation method. The calculations were based on the total number of test samples (*N* = 845), providing statistical insight into the reliability of the performance measures. The following subsections (3.1, 3.2, 3.3, and 3.4) will provide a detailed explanation of these steps.

### Dataset description and data preparation

**Fig. 2:**
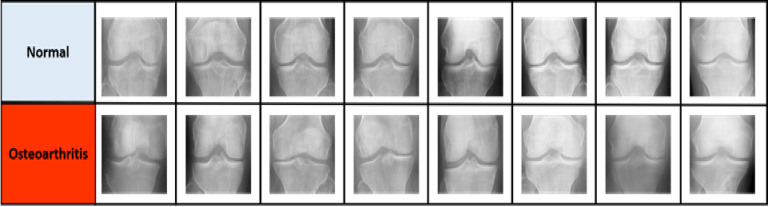
Samples of knee X-rays from the utilized dataset^50^.

The knee X-rays employed in this investigation to train the suggested framework were sourced via Kaggle^50^, consisting of the KOA-graded data set. The dataset was graded into two grades to differentiate the 3835 knee X-rays in total. Figure 2 displays samples of knee images from the utilized dataset. Competent professionals carefully evaluate each image in the collection to determine if it shows OA or normal. The total amount of data needed to train, verify, and test the CNN framework is displayed in Table 2. The images in the dataset were changed to 224 × 224 pixels in size to facilitate the model’s analysis due to their uniform size and match the input requirements of pre-trained CNN architectures. AlexNet’s images were reduced to 227 × 227 pixels to match its original architectural design and input layer arrangement. This preprocessing step follows recognized best practices in transfer learning to ensure complete compatibility with pre-trained weights and stable feature. While down sampling may theoretically reduce fine-grained detail, CNNs extract hierarchical features—early layers capture local textures, while deeper layers integrate broader structural patterns. Clinically relevant signs, such as osteophytes and joint-space constriction, are portrayed as structural patterns that remain unchanged upon resizing. Bilinear interpolation was employed to reduce aliasing and distortion. The dataset was scaled, normalized, and null entry removal applied to guarantee standardized and ready-made input data for further analytical steps.

**Table 2:** Distribution of the utilized dataset.

Group	Grade	Number of images
Train	Normal	810
	Osteoarthritis	1540
Validation	Normal	210
	Osteoarthritis	430
Test	Normal	569
	Osteoarthritis	276

### Feature extraction

The next stage after pre-processing data is FE. The TL procedure trains three pre-trained frameworks: Alex-Net, VGG19Net, and Google-Net. Our scenario’s objective is to classify medical images by pre-trained DLs. Consequently, we must use an adequately chosen knee orthosis dataset to train the network. Since not all models had the same initial size, the image resize process was applied to ensure all images fit the model’s initial size. The retrieved feature set is then filtered to contain particular characteristics that establish whether or not the afflicted person has the KOA. A brief description of each network is presented below.

**Fig. 3:**
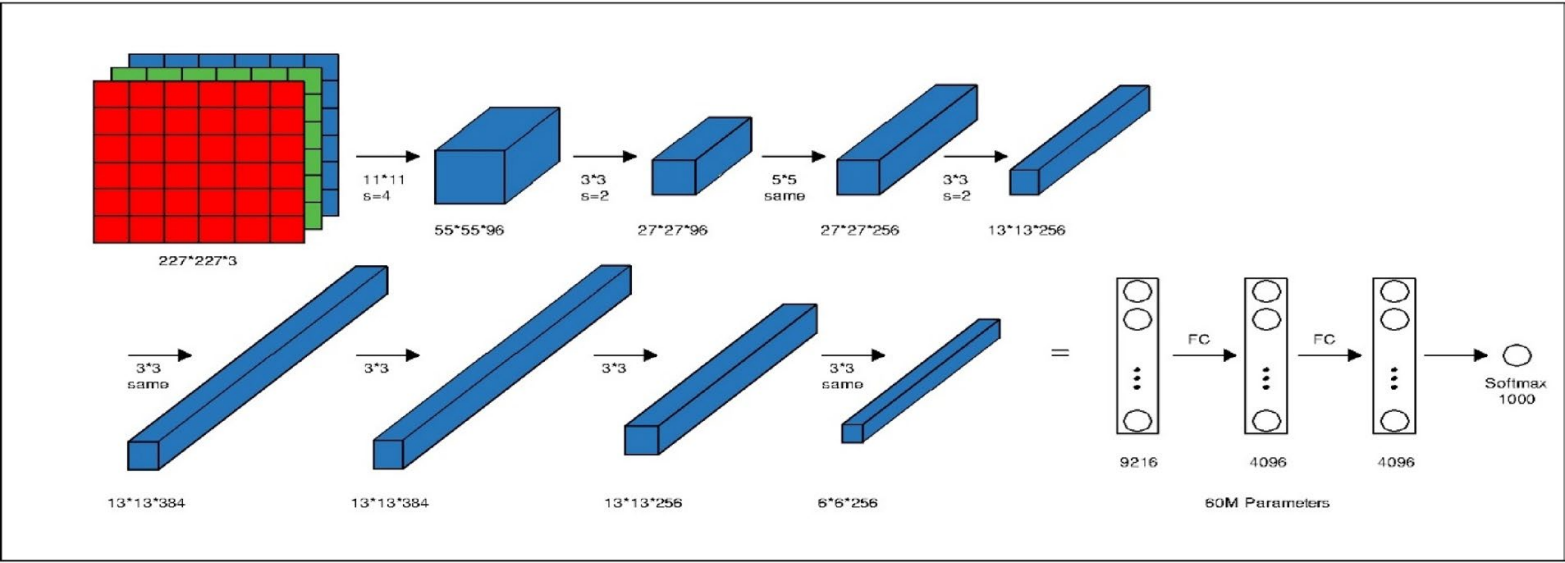
Alexnet architecture.

AlexNet is one of the earliest deep convolutional neural networks that demonstrated the effectiveness of deep learning in large-scale image classification. It consists of stacked convolutional and pooling layers followed by fully connected layers, and it introduced techniques such as ReLU activation and dropout to improve training efficiency and reduce overfitting, as shown in Fig. 3. Its relatively simple and shallow architecture makes it suitable as a baseline model for extracting general visual features.

**Fig. 4:**
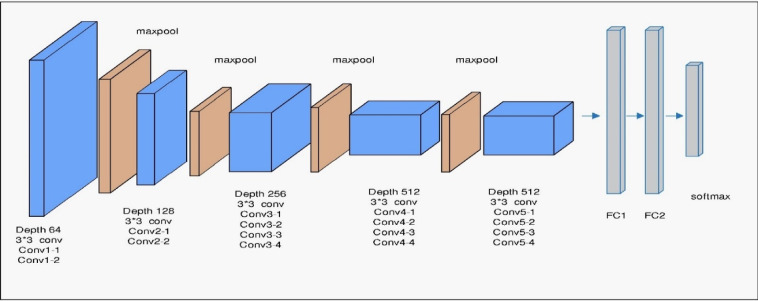
VGG19 architecture.

VGG19 is a deeper convolutional neural network characterized by a uniform architecture that uses small (3 × 3) convolutional filters stacked sequentially to increase depth and representational power. With 19 weight layers (see Fig. 4), it captures more detailed and hierarchical image features compared to earlier networks. Its straightforward and consistent design makes it effective for transfer learning and feature extraction tasks.

**Fig. 5:**
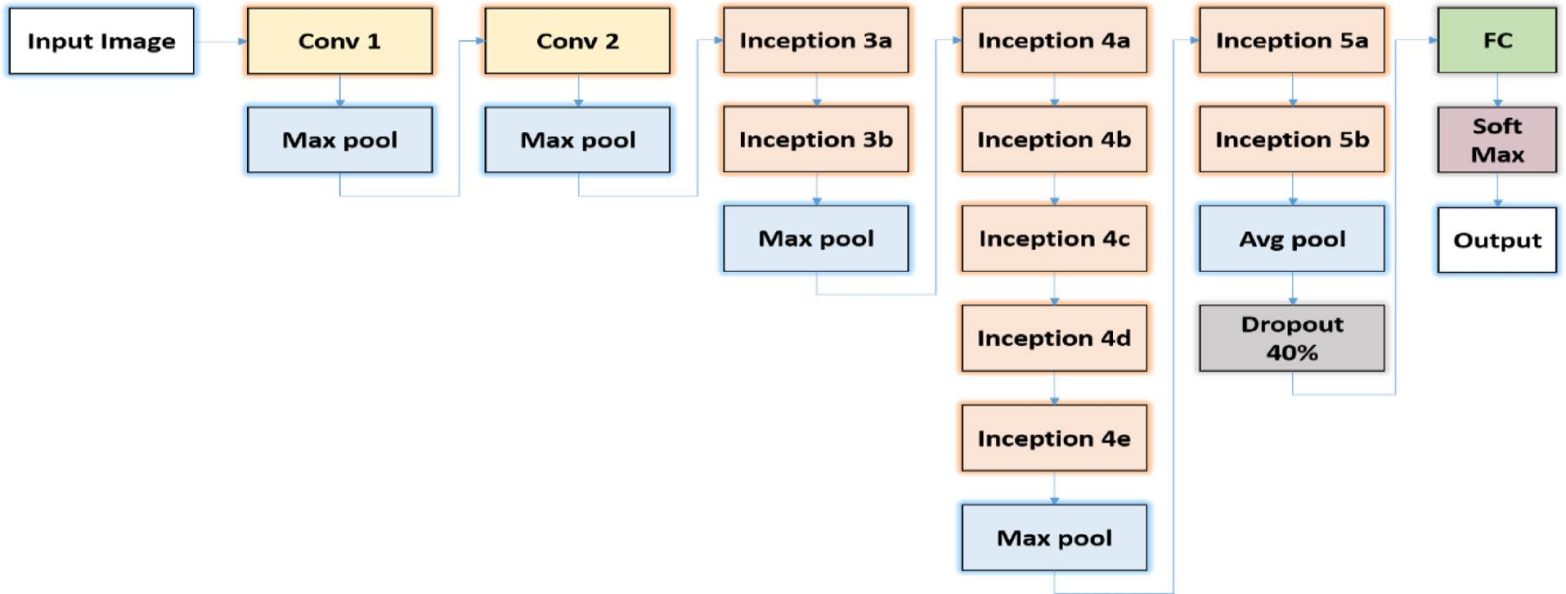
GoogleNet architecture.

GoogleNet, also known as Inception-v1, introduces the inception module, which applies multiple convolutional filters of different sizes in parallel within the same layer. This design allows the network to capture multi-scale features efficiently while keeping computational cost relatively low. Its architecture is deeper and more computationally efficient than earlier models, making it powerful for extracting rich and diverse feature representations (Fig. 5).

### Feature selection

FS is an approach adopted to decrease the obtained features from X-rays. More linked features increase the accuracy of categorization. Consequently, feature selection was applied to GoogleNet features to reduce dimensionality and retain the most discriminative information for KOA classification. This approach minimizes redundancy from less informative features in the other networks while preserving clinically relevant patterns. Eleven binary-form optimizers were trained and compared to choose the best optimizer: BER, HHO, JAYA, SBO, GSA, SFS, MVO, BBO, WOA, PSO, and TSH.

#### Al-Biruni earth radius algorithm

BER^24^ optimization approach, inspired by the calculation of an earth’s radius, is used for estimating the area of search solutions in the cooperative behavior of swarm members to achieve their worldwide objectives. BER seeks to strike a balance between ensuring rapid convergence and minimizing the stabilization of local optimums. This is done by implementing the BER approach, which assists in enhancing exploitation behavior, establishing a proper balance of exploration and exploitation, broadening search space exploration, and increasing variation within current population members. Preliminary findings indicate that BER is competitive, promising, and can surpass existing swarm optimization-based evolutionary approaches.

Members of groups are frequently split up into smaller groups to do different activities at distinct times and work together to attain their objectives, which inspired the idea of BER. Exploration and exploitation are typically used to determine the optimum answer to an optimization challenge. In our scenario, BER separates individuals into two subgroups, each responsible for either of the two tasks. Exploration and exploitation tasks in BER guarantee a comprehensive examination of the search space, avoiding the stagnation of local optima. Since most cooperative optimization strategies desire everyone to participate in exploitation after iterations, local optima may stagnate. By maintaining a group of search agents who are constantly exploring new regions of the search space, BER is able to prevent this type of scenario. The BER also rapidly expands the number of researchers in the search space if the algorithm’s performance doesn’t change after three cycles of solution mutation.

##### Fundamental concepts and formula

Optimization algorithms seek the most effective solution to solve an issue given limitation. A member of the population may be depicted as a vector in BER, $\:\overrightarrow{S}=\left\{{S}_{1},\:{S}_{2},\:\dots\:,\:{S}_{d}\right\}\:\in\:\:{R}_{d}$, where $\:{S}_{i}$ is one of the optimization problem’s parameters, and d is the search space’s size. f is a fitness function employed in the suggested technique to assess an individual’s performance up to a specific point. For the population searching for a certain optimal vector S* that maximizing the fitness function, this technique consists of the following stages. The process begins with a set of random solutions. To begin the optimization process, BER requires the following parameters: population size, dimension, lower and upper bounds for each solution, and the fitness function.

##### Proportion of exploration to exploitation

The procedure divides the population into subgroups and dynamically modifies the numeral of individuals in all the groups to enhance the ratio of exploration to exploitation activities. The procedure begins by splitting the population into 2 groups: exploration and exploitation. 30% of the population is in the exploitation group, while 70% is in the exploration group. To enhance the fitness values of individuals in each group, the number of participants in the exploitation task is first set at 30% and then gradually increased throughout the optimization iterations to 70% of the population count. On the other hand, the initial numeral of members in the exploration group is set at 70%, and over iterations, the numeral is decreased to 30%. The global average of individual fitness can rise more significantly using this approach. Furthermore, the elitism method guarantees that the population’s process converges, which involves retaining the leading solution for the process if no superior one is found. In the BER optimization process, if a solution’s fitness does not increase much after 3 rounds, it may be an optimal locale, and the mutation procedure can be used to create additional exploratory individuals.

##### Exploration operation

Exploration is responsible for avoiding local optima stagnation and identifying intriguing areas in the search space by moving towards the optimal answer, as explained later.

- Heading towards the best solution.

In the exploration group, an individual employs this technique to look for prospective regions within the search space surrounding its current location. Finding a superior alternative in terms of fitness value is accomplished by consistently searching over the available possibilities. For this, the BER search requires the use of the following equations:

1 $$r = h~\frac{{\cos \left( x \right)}}{{1 - \cos \left( x \right)}}~$$

2 $$\vec{D} = ~\overrightarrow {{r_{1} }} .\left( {\vec{S}\left( t \right) - 1} \right)$$

3 $$\vec{S}\left( {t + 1} \right) = ~\vec{S}\left( t \right) + ~\vec{D}.\left( {2\overrightarrow {{r_{2} }} - 1} \right)~$$

Where h is a randomly chosen numeral from the range [0, 2], $\:0<x\le\:180$, $\:\overrightarrow{S}\left(t\right)$ is the solution vector at iteration $\:t$. $\:\overrightarrow{D}$ is the circle’s diameter. Thus, the search agent will explore promising regions, and $\:\overrightarrow{{r}_{1}}$ and $\:\overrightarrow{{r}_{2}}$ are coefficient vectors and Eq. 3 determines their values.

##### Exploitation operation

Upgrading existing solutions is the responsibility of the exploitation team. At each cycle, the BER determines the individual’s fitness values and chooses the best one. As shown in the following subsections, the BER deploys two distinct strategies to accomplish exploitation.

- Heading towards the best solution.

The search agent is guided towards the optimal solution by the equations below:

4 $$\vec{S}\left( {t + 1} \right) = r^{2} ~\left( {\vec{S}\left( t \right) + ~\vec{D}} \right)~$$

5 $$\vec{D} = ~\overrightarrow {{r_{3} }} \left( {\vec{L}\left( t \right) - \vec{S}\left( t \right)} \right)~$$

Where $\:\overrightarrow{S}\left(t\right)$ is the solution vector at iteration $\:t,$ $\:\overrightarrow{D}$ is the vector of distance, $\:\overrightarrow{{r}_{3}}$ is a random vector which regulates the stages leading to the optimal solution and is computed using Eq. 3, and $\:\overrightarrow{L}$ is the best solution vector.

b. Investigating areas around the best solution.

The most promising location is the one surrounding the best solution (leader). As a result, some people seek the best option to discover a better one. The BER performs this procedure utilizing the following equation.

6 $$\vec{S}\left( {t + 1} \right) = r~\left( {\overrightarrow {{S*}} \left( t \right) + ~\vec{k}} \right)~$$

7 $$\vec{k} = z + ~\frac{{2~ \times t^{2} }}{{N^{2} }}$$

**Fig. 6:**
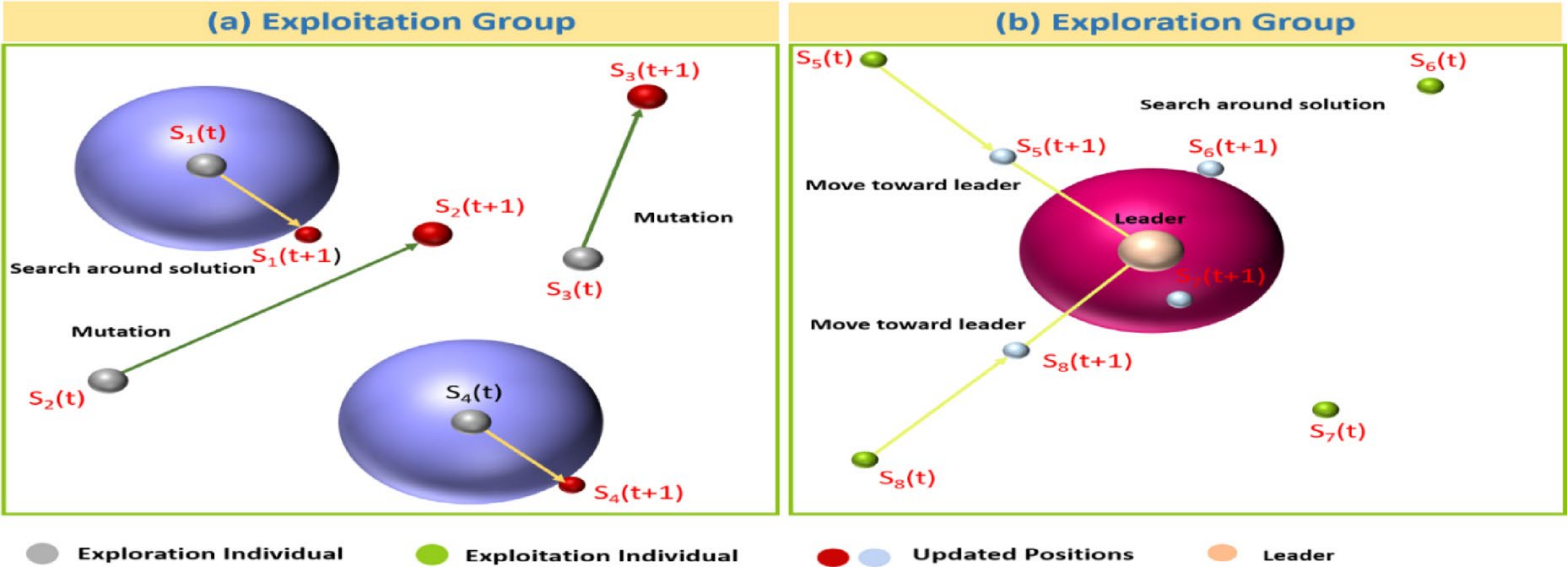
Exploration and exploitation-related activities.

Where $\:\overrightarrow{S\mathrm{*}}$ is the optimal solution, z is a random numeral within the range [0, 1], t is the iteration numeral, and N is the overall numeral of iterations. Figure 6 illustrates the exploration and exploitation attempts.

##### Mutation operation

The BER’s excellent exploration potential is mainly due to the mutation, a genetic operator that creates and sustains population variety. One or more components in individuals may be disrupted in a probabilistic local random manner, preventing early convergence and avoiding local optima. Another intriguing subject is introduced by this change in the search area.

8 $$\vec{S}\left( {t + 1} \right) = ~\vec{k}*z^{2} ~ - h\frac{{\cos \left( x \right)}}{{1 - \cos \left( x \right)}}$$

##### Choosing the best solution

**Algorithm 1:**
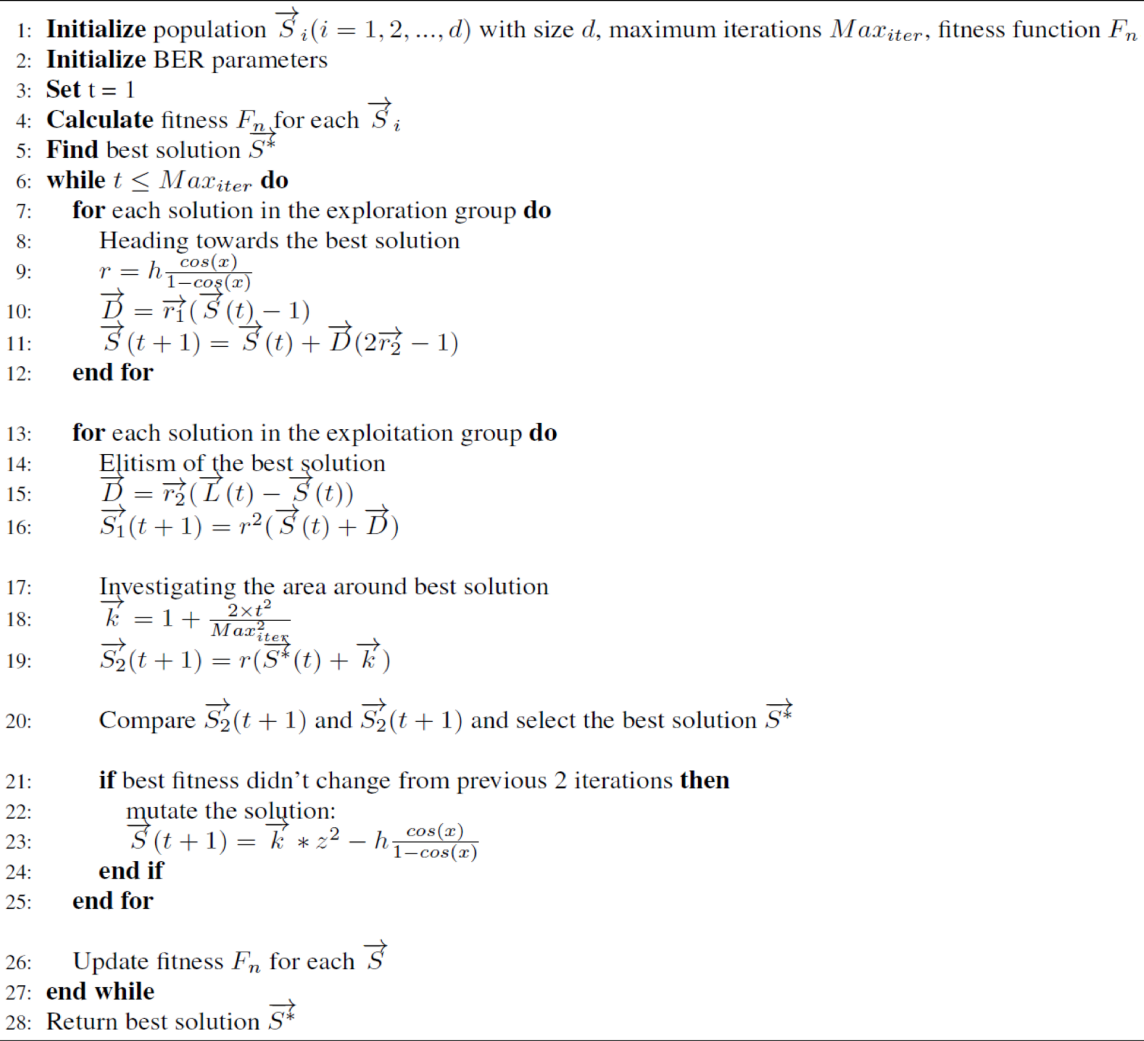
The proposed Al-Biruni earth radius (EER) based optimization algorithm.

**Fig. 7:**
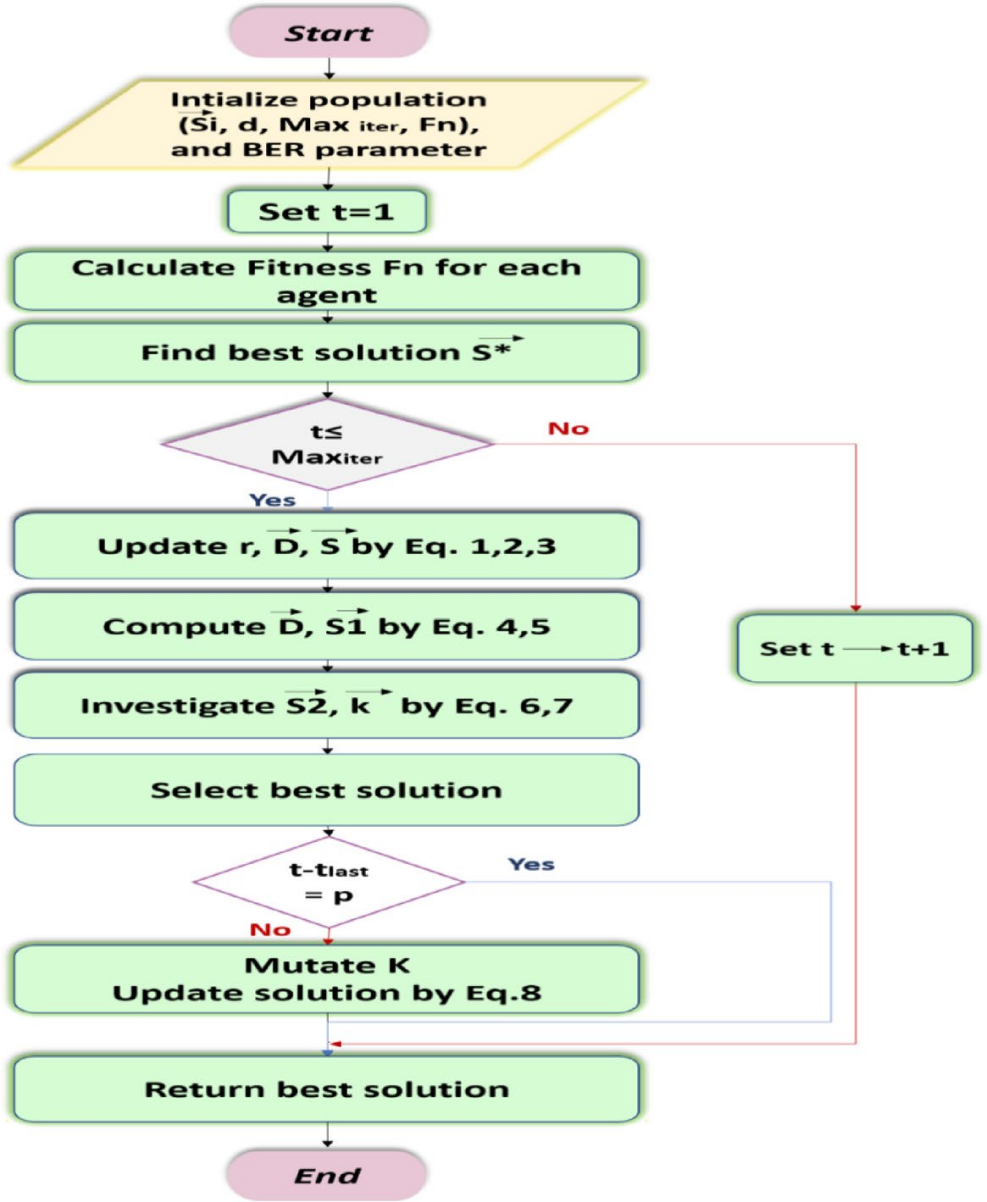
The flowchart of BER.

The BER selects the best solution for the following iteration to guarantee the quality of the identified solutions. Although the elitism strategy increases system efficacy, it may hasten the convergence of multimodal functions. It should be noted that because the BER use a mutation method to find members of the exploration group, it possesses extraordinary exploratory abilities. The BER’s powerful exploration capabilities allow it to avoid early convergence. Algorithm 1 displays the BER pseudocode and Fig. 7 indicates the flowchart of the BER algorithm. First, we provide specific input parameters to the BER, such as the numeral of iterations, population size, and mutation rate. During the search rounds for the optimum solution, the BER procedure dynamically modifies the group’s participation count. To fulfill its tasks, each group adopts two separate tactics. After every cycle, the BER ranks solutions randomly to guarantee diversity and exploration. In one cycle, for instance, a solution from the exploration group may join the exploitation group later. The BER’s elitism strategy helps to prevent losing the leader between iterations.

### Classification

Two fundamental ML models were applied and tested – MLP and LSTM network to classify the selected features.

#### Long short-term memory (LSTM)

**Fig. 8:**
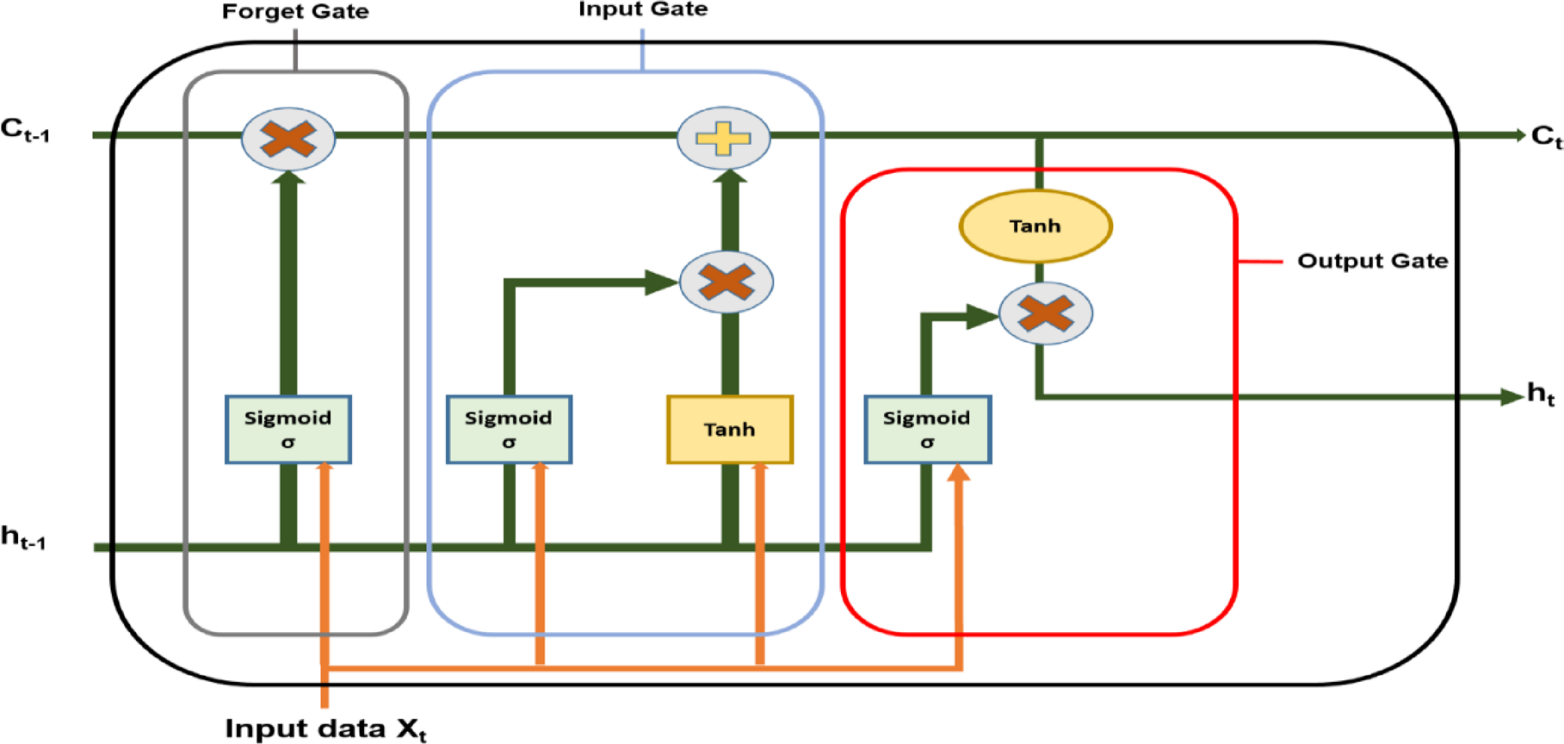
The structure of a single LSTM cell with gates.

A specific type of conventional ANN that uses feedback loops to record recent input events as activations is called an RNN. The RNN is among the most advanced approaches for categorization issues involving sequential data, thanks to the advancement of DL techniques in recent years. However, while the RNN can learn any length, it has drawbacks such as gradient disappearing and explosion^51–53^. Hochreiter and Schmidhuber presented an instance of RNN that overcomes this by replacing the RNN cell with a gated cell called the LSTM network. LSTM is an RNN widely used, particularly in sequential forecasting. When processing a succession of datasets, such as discharge time series, and managing all the data points, this unique feature of LSTM is utilized separately. Figure 8 shows the fundamental architecture of a single LSTM network. The key to processing and remembering long-term information is LSTM feedback connections, which distinguishes it from the conventional feedforward neural network. LSTM technology is frequently used in natural language processing applications, such as text categorization and translation. It also has applications in various engineering disciplines, including fault detection, predictive maintenance, and process control. The LSTM algorithm’s capacity to learn through time series data while still processing static information renders it valuable in the energy, engineering, and robotics sectors.

The memory cell in an LSTM network, represented by C_t_, has self-loops that retain the temporal information in the cell state. Three gates control information flowing in the network: forget gate f_t_ $\in$ [0; 1], Input gate i_t_ $\in$ [0; 1], and output gate O_t_ $\in$ [0; 1]. In order to reduce misclassification rates, the network learns throughout training what needs to be committed to memory and when to turn on reading and writing. The Forget gate explicitly determines which data from the prior memory cell state needs to be erased since it has expired. By selecting pertinent data from the candidate memory cell state, the input gate modifies the cell state $\:{\mathrm{C}}_{\mathrm{t}}^{\mathrm{*}}$. Table 3 shows the equations for each cell, which derive the value of each gate, C_t_, and output y_t_ of the network. The output gate guarantees the system only examines the most important information for the forecast function by filtering the data from the memory cell^54^.

**Table 3:** Equations for all cells and gate operation for the LSTM network.

LSTM Component	Formula
Forget gate	f_t_ = sigmoid (W_f_ [y_(t−1)_, X_t_] + b_f_)
Input gate	i_t_ = sigmoid (W_i_[y_(t−1)_, X_t_] + b_i_)
Output gate	O_t_ = sigmoid (W_O_[y_(t−1)_, X_t_] + b_O_)
Cell state (memory cell value)	C_t_ = C_(t−1)_. f_t_ + $\:{\mathrm{C}}_{\mathrm{t}}^{\mathrm{*}}$.i_t_
Candidate for cell state	$\:{\mathrm{C}}_{\mathrm{t}}^{\mathrm{*}}=$sigmoid (W_C_[y_(t−1)_, X_t_] + b_C_)
Output	y_t_ = O_t_ ∗ tanh (C_t_)

W _[i, f, c, o]_ represents the weighted matrices, and b _[i, f, c, o]_ represents the bias vectors of the network. The LSTM model was trained on the selected features utilizing binary Al-Biruni earth radius (bBER) of the present signal under various operating scenarios, including standard, LG, LL, LLG, LLLG, and HIF. Training data is produced by altering fault resistances during LG, LL, LLG, and LLLG types of faults. The LSTM unit uses sigmoid and hyperbolic tangent functions for nonlinear activation and state updates.

Although LSTM networks were originally intended for temporal sequence modelling, its internal gating dynamics can be understood more broadly as a structured nonlinear transformation capable of modelling ordered dependencies inside high-dimensional representations. In the current approach, the deep feature vector derived from CNN backbones is transformed into an ordered feature sequence. This reformulation allows the LSTM to function as a progressive dependency encoder rather than a temporal predictor. Unlike traditional feedforward classifiers, which use a single global transformation, LSTM incorporates a recurrent state transition mechanism that allows for hierarchical refining of feature components via gated information flow. From an optimisation standpoint, the memory cell serves as an adaptive information accumulator, retaining discriminative activations while suppressing redundant or noisy components. This dynamic filtering approach significantly improves representational compactness prior to final categorization. As a result, LSTM can be considered as a learnable feature interaction regulator capable of capturing higher-order correlations that would otherwise be implicit in static multilayer perceptron mappings.

This behavior is theoretically justified by Sepp Hochreiter and Jürgen Schmidhuber’s original LSTM formulation, which included the gated cell state to control gradient propagation and retain informative dependencies across extended computational steps. As a result, in this study, LSTM is used as a gated dependency learning mechanism over structured feature representations produced from deep convolutional encoders rather than for temporal modelling in general.

## Evaluation criteria

### Performance metrics for pre-trained models and classifiers

Utilizing the confusion matrix, metrics such as accuracy, precision, and F1-score can be determined by contrasting the anticipated labels to genuine ones. The confusion matrix divides into four categories: true positive (TP) value, true negative (TN) value, false positive (FP)value, and false negative (FN)value. Table 4 describes the evaluation metrics.

**Table 4:** The evaluation metrics used involve TP, TN, FP, and FN.

Metric& Abbreviation	Formula
Accuracy (ACC)	ACC *=* $\:\frac{\mathrm{T}\mathrm{P}+\mathrm{T}\mathrm{N}}{\:\mathrm{T}\mathrm{P}+\mathrm{F}\mathrm{N}+\mathrm{T}\mathrm{N}+\mathrm{F}\mathrm{P}}$
Sensitivity (SENS)	SENS *=* $\:\frac{\mathrm{T}\mathrm{P}}{\mathrm{T}\mathrm{P}+\mathrm{F}\mathrm{N}}$
Specificity (SPEC)	SPEC *=* $\:\frac{\mathrm{T}\mathrm{P}}{\mathrm{T}\mathrm{N}+\mathrm{F}\mathrm{P}}$
Positive predictive value (P-value)	P-Value *=* $\:\frac{\mathrm{T}\mathrm{P}}{\mathrm{T}\mathrm{P}+\mathrm{F}\mathrm{P}}$
Negative predictive value (N-value)	N-Value *=* $\:\frac{\mathrm{T}\mathrm{N}}{\mathrm{T}\mathrm{N}+\mathrm{F}\mathrm{N}}$
F1-score	F1-score *=* $\:\frac{2\mathrm{T}\mathrm{P}}{2\mathrm{T}\mathrm{P}+\mathrm{F}\mathrm{P}+\mathrm{F}\mathrm{N}}$

### Performance indicators for the optimizers

The effectiveness of the proposed method in choosing features for evaluation is evaluated through trials using the following metrics (see Table 5). Where is the numeral of repetitions, ∗ is the best solution, is the overall numeral of points, is a point’s class, is the classifier’s output, _ℎ_ shows the extent to which the two inputs match, $\:{\mathrm{g}}_{\mathrm{j}}^{\mathrm{*}}$ is a vector size, and is dataset size.

**Table 5:** Experiments were conducted to determine how successfully the proposed optimizers choose features for evaluation utilizing evaluation measures.

Metric	Formula
Average Error	Avg. Error = 1 - $\:\frac{1}{M}$ $\:\sum\:_{j=1}^{M}\frac{1}{N}\:\sum\:_{i=1}^{N}\mathrm{M}\mathrm{a}\mathrm{t}\mathrm{c}\mathrm{h}(\mathrm{C}\mathrm{i},\:\mathrm{L}\mathrm{i})$
Average Select-Size	Avg. Select Size $\:=\:\frac{1}{M}\:\sum\:_{j=1}^{M}\frac{\mathrm{S}\mathrm{i}\mathrm{z}\mathrm{e}\left({g}_{j}^{\mathrm{*}}\right)}{D}$
Best Fitness	Best Fitness = $\:{Min}_{j=1}^{M}{g}_{j}^{\mathrm{*}}$
Worst Fitness	Worst Fitness = $\:{Max}_{j=1}^{M}{g}_{j}^{\mathrm{*}}$
Standard Deviation (SD)	SD= $\:\sqrt{\frac{1}{M-1}\:\sum\:{({g}_{j}^{\mathrm{*}}-Mean)}^{2}}$
Mean	Mean =$\:\frac{1}{M}\:\sum\:_{j=1}^{M}{g}_{j}^{\mathrm{*}}$

## Results and discussion

### Feature extraction results

Metrics involving as F1-score, N-Value, P-Value, sensitivity, specificity, accuracy and time are employed to assess the effectiveness of extracted features. The FE method effectively identified the most crucial attributes for the categorizing task if the extracted features exhibit improved accuracy, sensitivity, specificity, F1-score, a low P-value, and Time (Refer to Table 6). The Google-Net DL model was utilized to extract features in this investigation, resulting in an accuracy of 0.92169016%. The results in Table 6 demonstrate that the feature obtained from Google Net surpasses other deep NN. This degree of performance implies that Google-Net can accurately recognize and integrate the most beneficial characteristics from the supplied dataset, a critical capability for handling the picture classification challenge.

**Fig. 9:**
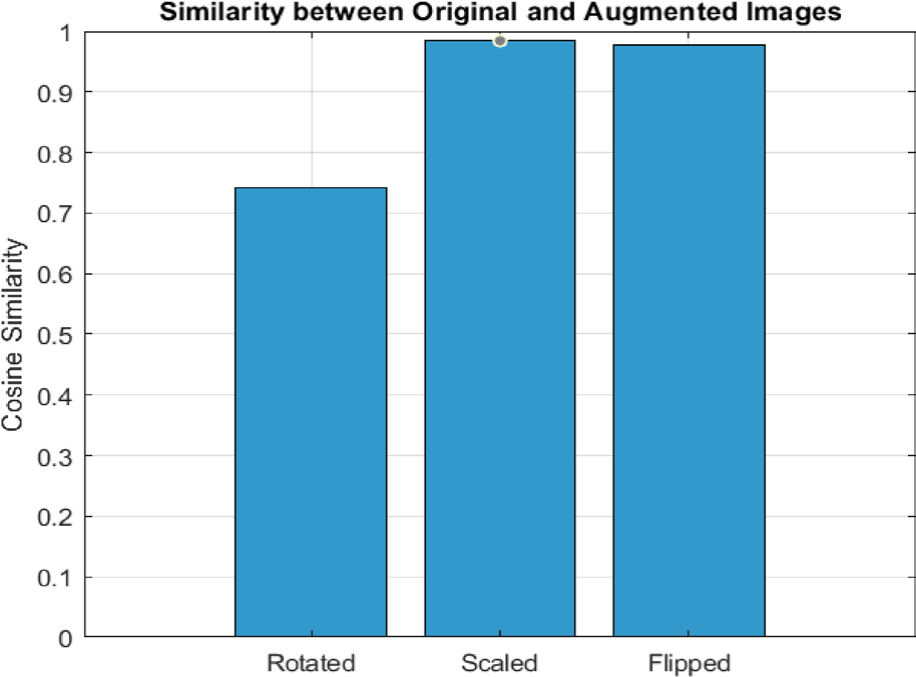
Cosine similarity of original vs. augmented knee X-rays (Rotated, Scaled, Flipped).

To test Google-Net feature representations for geometric variations, we rotated (± 15°), scaled (± 10%), and horizontally flipped knee X-ray images. The cosine similarity between the original and augmented feature vectors (Fig. 9; Table 7) revealed that scaling and flipping maintained high similarity (0.985 and 0.978), whereas rotation lowered similarity to 0.742, showing modest sensitivity to orientation alterations. Rotation reduces similarity, whereas scaling and flipping maintain feature invariance. These findings highlight the need of rotation-aware augmentation for improving feature invariance in KOA classification.

**Table 6:** Assessing the characteristics obtained by CNN deep NN.

Models	Accuracy	Sensitivity (TPR)	Specificity (TNR)	*P*-value (PPV)	*N*-value (NPV)	F-score	Time(S)
Alex-Net	0.885931868	0.694287609	0.973023881	0.8514518	0.894512329	0.732085714	631.3418
VGG19-Net	0.890617955	0.736382216	0.968619672	0.817833877	0.907370552	0.759554386	553.9418
Google-Net	0.92169016	0.753094118	0.973023881	0.8517822	0.939990202	0.79905397	428.4418

**Table 7:** Cosine similarity between original and augmented knee X-rays using GoogleNet features.

Augmentation technique	Cosine Similarity
Rotated	0.74192
Scaled	0.98471
Flipped	0.97775

### Feature interpretation

**Fig. 10:**
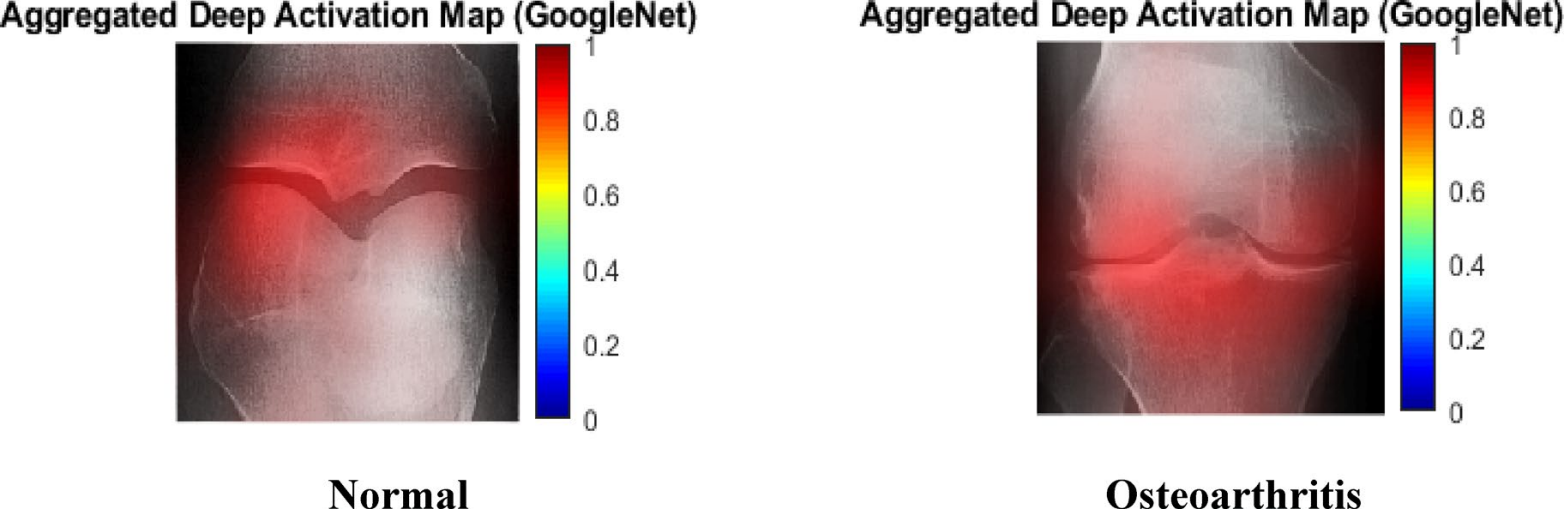
Aggregated deep activation maps from the final convolutional layer of Googlenet for representative Normal and KOA cases.

To improve interpretability, activation maps from deep convolutional layers were combined and overlaid on input radiographs. The heatmaps generated showed intense reactions along the tibiofemoral joint region and neighboring bone shapes. These anatomical regions have a direct relationship to KOA grading criteria, demonstrating that the learned deep features represent clinically important structural patterns. Figure 10 depicts deep activation maps from GoogleNet’s last convolutional layer for the Normal and KOA scenarios. The KOA image indicates stronger and more localised activation along the tibiofemoral joint space and marginal bone regions, which correspond to radiographic features associated with osteoarthritis severity.

### Feature selection results

This investigation utilized sophisticated FS approaches to apply, train and compare eleven different algorithms in binary variants: BER, HHO, JAYA, SBO, GSA, SFS, MVO, BBO, WOA, PSO, and TSH. Table 8 presents an exhaustive analysis of the comparative performance of different strategies, outlining the outcomes achieved using each methodology. The results show that the suggested bBER approach is streamlined and regularly beats its competitors in key performance parameters, as indicated by its domination above other binary FS methods.

**Table 8:** The assessment of the recommended FS strategy by bBER in contrast to other competing approaches.

Optimizer Metric	bBER	bHHO	bJAYA	bPSO	bTSH	bWOA	bBBO	bMVO	bSBO	bSFS	bGSA
Average error	**0.79469**	0.81189	0.85119	0.84569	0.85529	0.84549	0.81389	0.82239	0.85399	0.83199	0.82549
Average Select size	**0.74749**	0.94749	0.80808	0.94749	0.80868	0.811089	0.81113	0.80439	0.91177	0.87029	0.88989
Average Fitness	**0.85789**	0.87409	0.88239	0.87249	0.89539	0.88029	0.87819	0.90219	0.91219	0.88019	0.88549
Best Fitness	**0.75969**	0.79439	0.83589	0.85279	0.78509	0.84439	0.86789	0.82739	0.85529	0.85799	0.78879
Worst Fitness	**0.85819**	0.86129	0.94589	0.92049	0.88669	0.92049	0.95439	0.94539	0.93499	0.93419	0.90389
Standard deviation Fitness	**0.68019**	0.68489	0.70309	0.68429	0.69419	0.68649	0.72919	0.73499	0.74519	0.68549	0.68649

The result of our proposed optimizer are in bold.

**Fig. 11:**
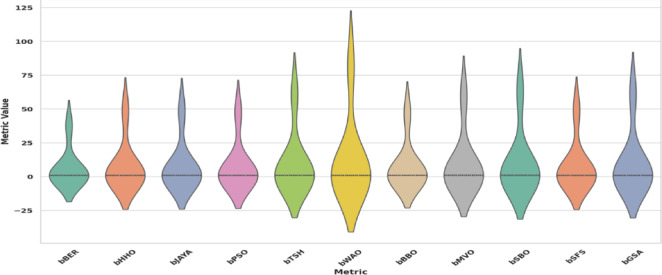
The performance of the violin plot algorithm for bBER and other algorithms.

Figure 11 compares the violin plots of algorithm performance for bBER, bHHO, bJAYA, bSBO, bGSA, bSFS, bMVO, bBBO, bWOA, bPSO, and bTSH. A violin plot employs density curves to illustrate numerical data distributions for multiple categories. The breadth of each curve shows the estimated frequency of data points in every location. Densities are commonly supplemented by an overlay chart type, like a box plot, to offer extra information. As indicated from this figure that bBER beats peer methods in both accuracy and consistency, as indicated by the left-shifted and tight violin distributed.

**Fig. 12:**
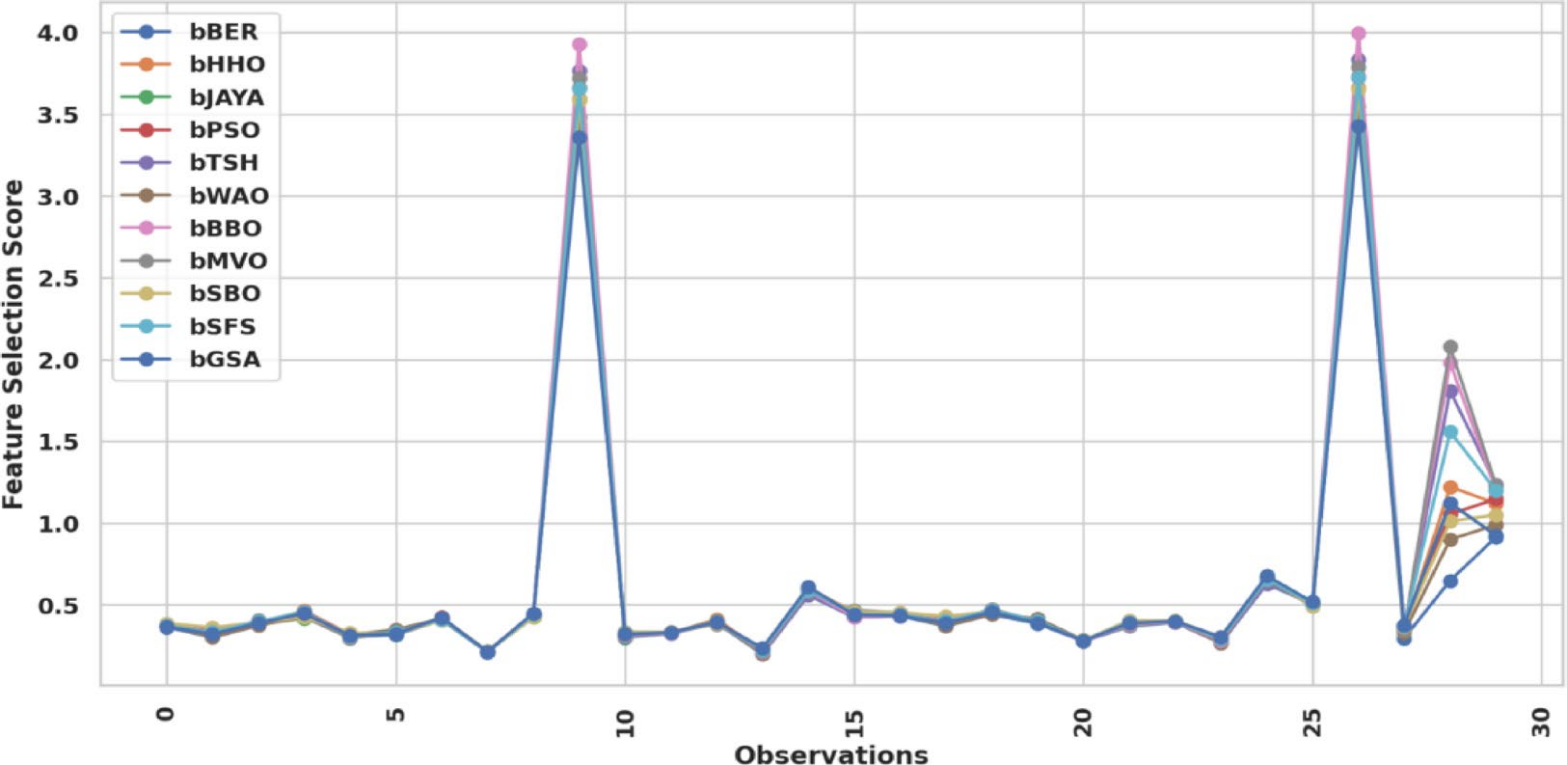
Trend of FS scores of the bBER procedure in contrast to alternative techniques.

Figure 12 shows the trend of FS scores across bBER, bHHO, bJAYA, bSBO, bGSA, bSFS, bMVO, bBBO, bWOA, bPSO, and bTSH, demonstrating performance fluctuations. This Figure demonstrates how bBER remains robust, considering the trade-off among the size of the chosen set and fitness levels. The general pattern seen in the trend graphic lends credibility to this methodology. It emphasizes that bBER is not as intent on FS since it finds a decent balance, making it more suitable for applications that require exact NDVI estimations. The measures have smooth trend lines, strengthening the program’s trustworthiness and performance in various ecological prediction problems. The bBER routinely outperforms cutting-edge FS strategies. Its popularity indicates high application for dimensional reduction in ML processes.

**Fig. 13:**
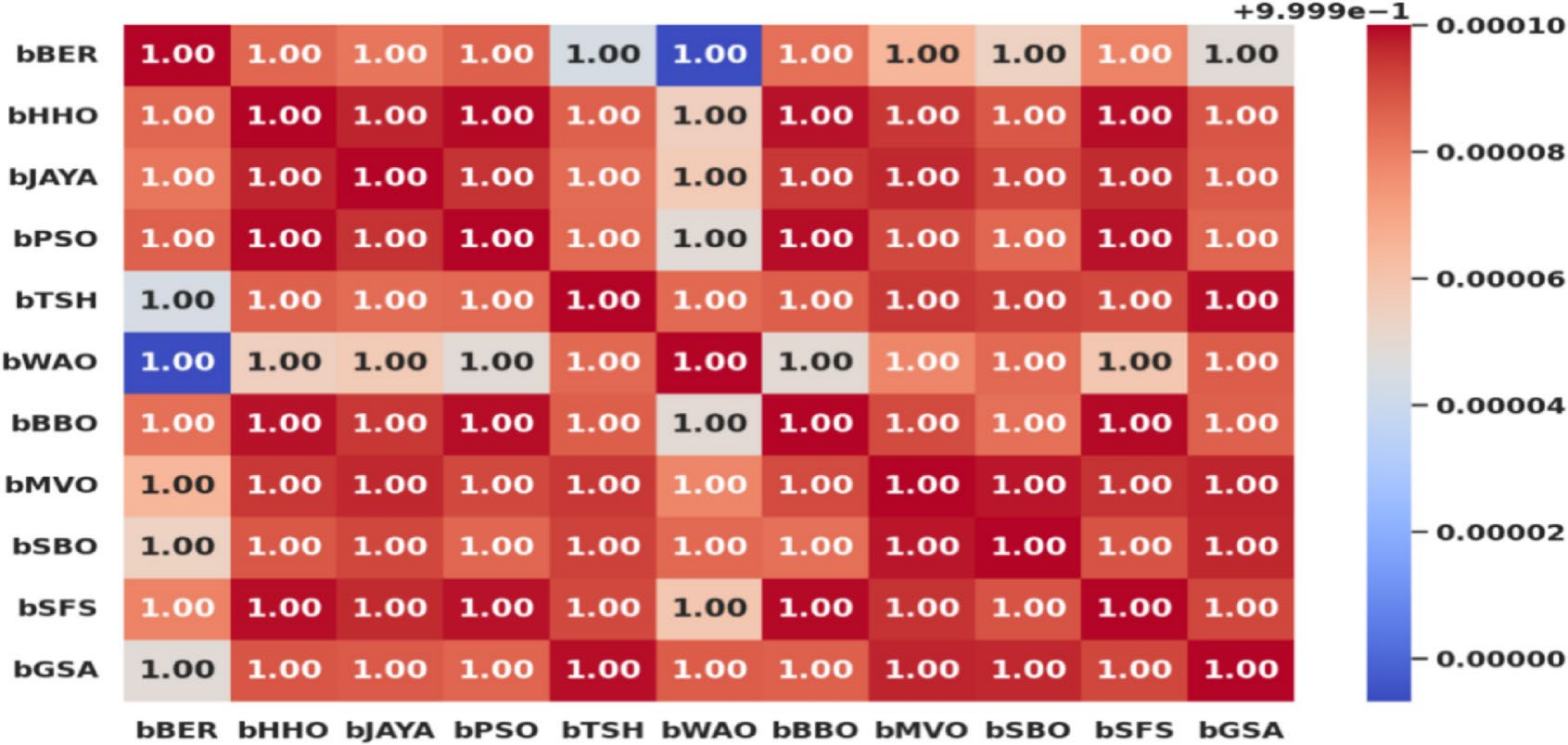
A correlation matrix between all optimization algorithms was utilized in this study.

Figure 13 shows the correlation matrix between all optimization algorithms was utilized in this study. To investigate the links, trends, and potential indicators in the data, the correlation matrix looks for components that exhibit positive or negative correlations.

**Fig. 14:**
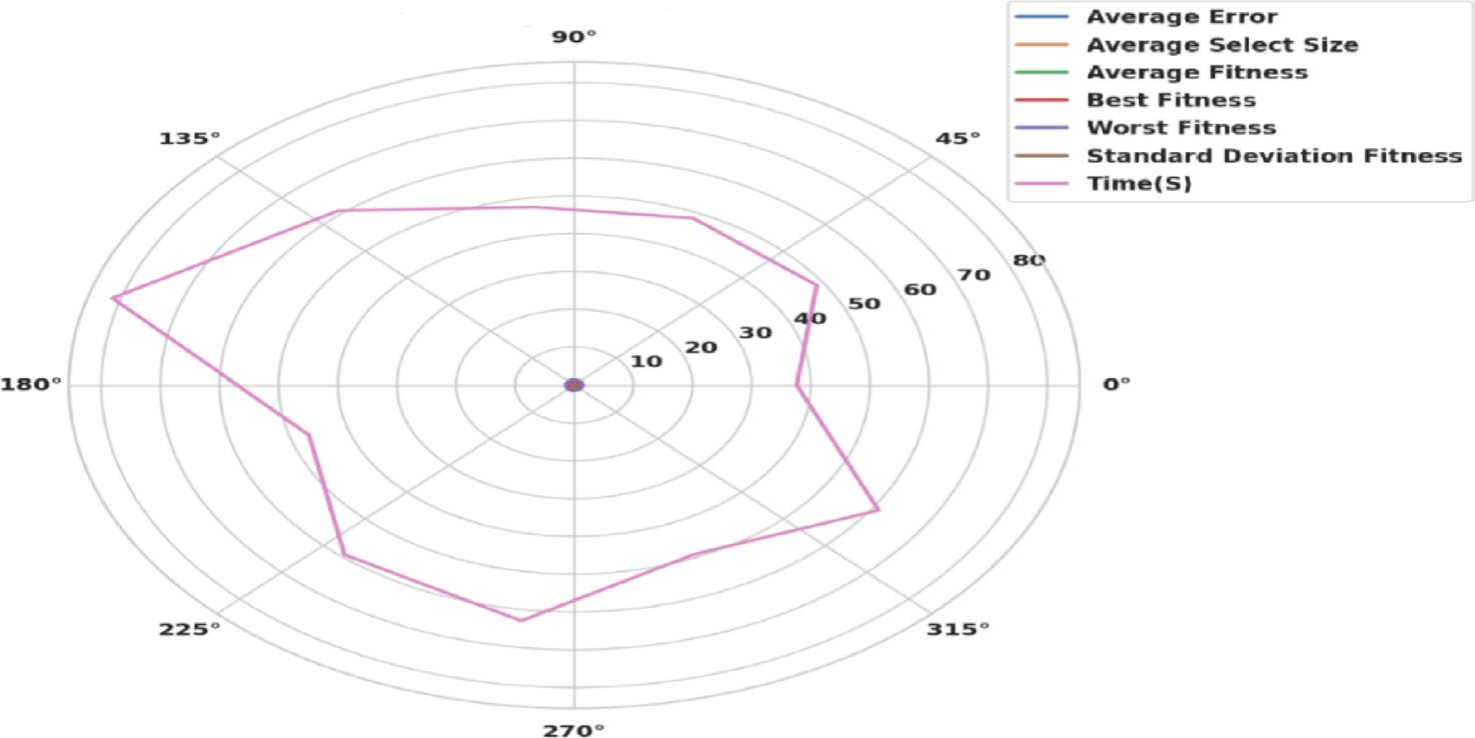
Radar Plot of BER performance.

Figure 14 shows a radar plot illustrating the performance of the BER algorithm. Multivariate data can be graphically displayed as a two-dimensional chart utilizing a radar plot, where more quantitative components are displayed on axes that start at the exact location. Although the axes’ relative location and angle are usually useless, the variables (axes) can be sorted into relative positions that show clear correlations, trade-offs, and plenty of additional comparative metrics employing a variety of heuristics, like procedures that plot data as the maximum total area. The radar plot displays BER’s adaptability by excelling in both solution quality (fitness) and efficiency (time) while retaining an equitable exploration-exploitation ratio. The symmetric shape emphasizes its suitability for a variety of optimization challenges.

**Fig. 15:**
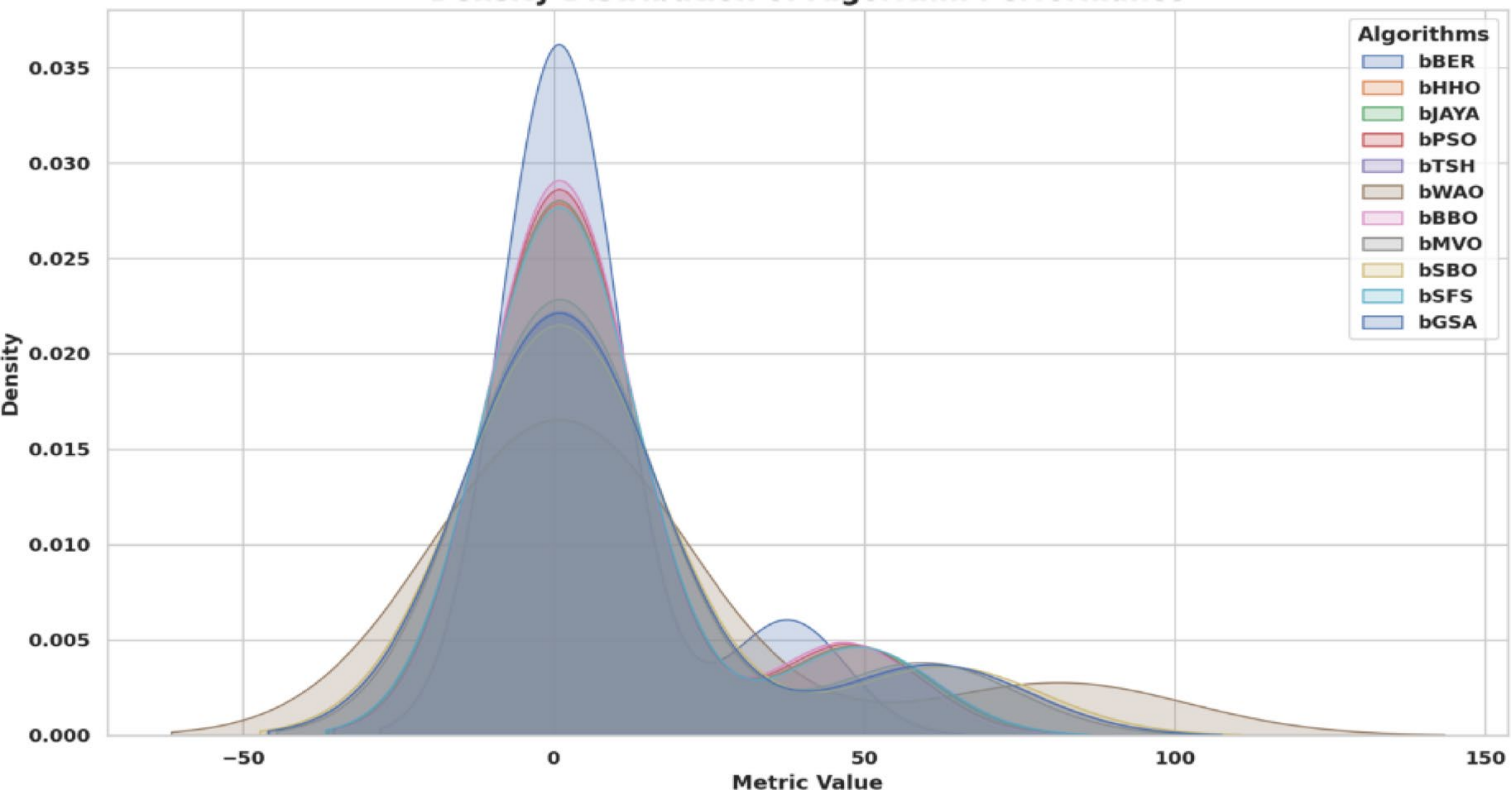
Density distribution of the bBER procedure in contrast to alternative techniques.

Figure 15 shows the density distribution of the bBER procedure in contrast to alternative techniques, the bBER technique’s noticeable effectiveness throughout the interval is highlighted by the graphical depiction which reached 0.037. This figure shows bBER’s supremacy, with its elevated, narrow density peak exhibiting greater performance and outstanding consistency in contrast to other approaches. This renders it an appealing candidate for optimization jobs that demand precision and dependability.

**Fig. 16:**
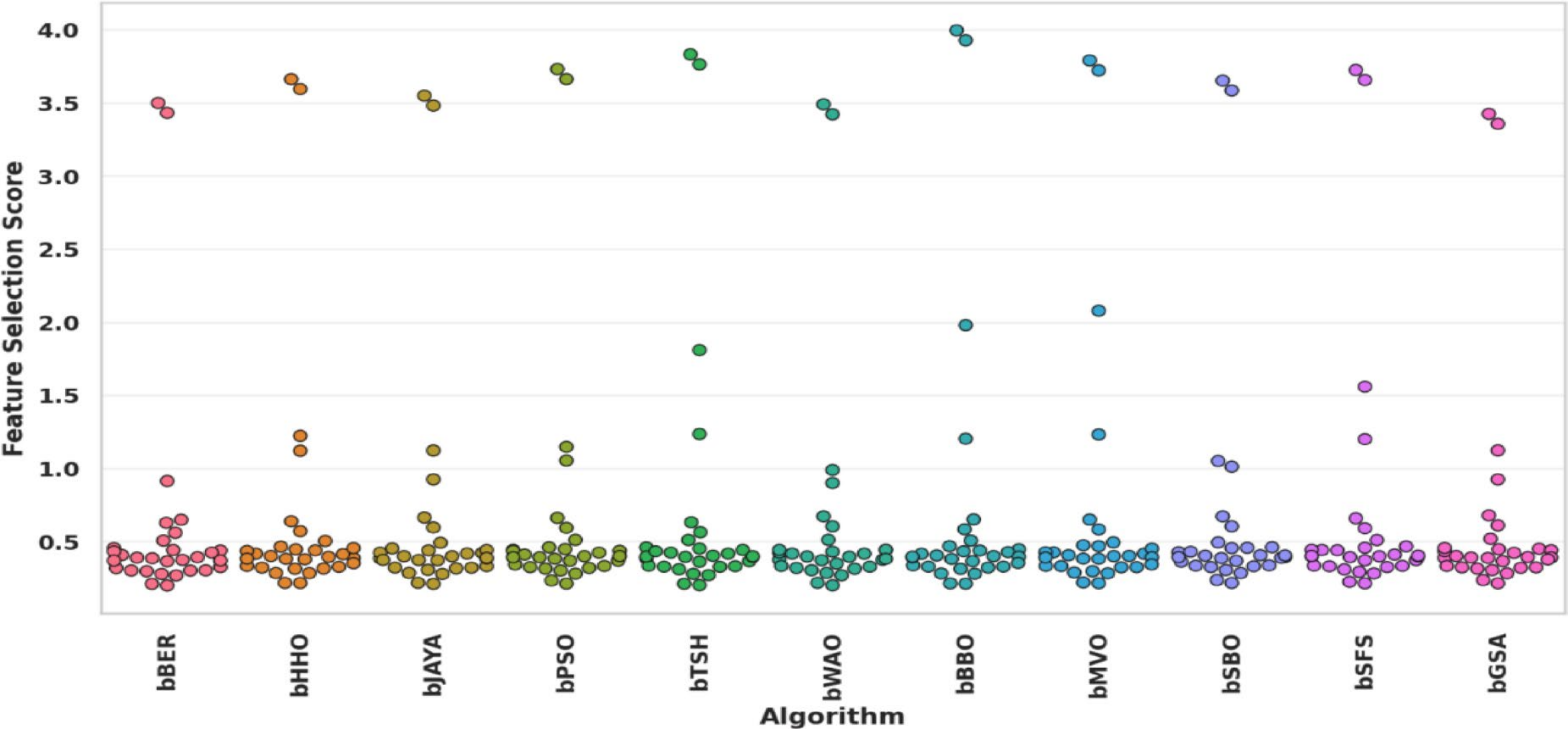
The Swarm plot of FS scores for the bBER algorithm compared to alternative techniques.

Figure 16 depicts a Swarm plot of FS scores for eleven different FS approaches, including the suggested bBER strategy. As indicated from this figure that bBER achieves the greatest and most consistent FS scores while exhibiting minimum performance fluctuation. Its dense cluster at the top scores stands out against competitors’ scattered distributions, establishing it as the most dependable alternative for FS tasks.

**Fig. 17:**
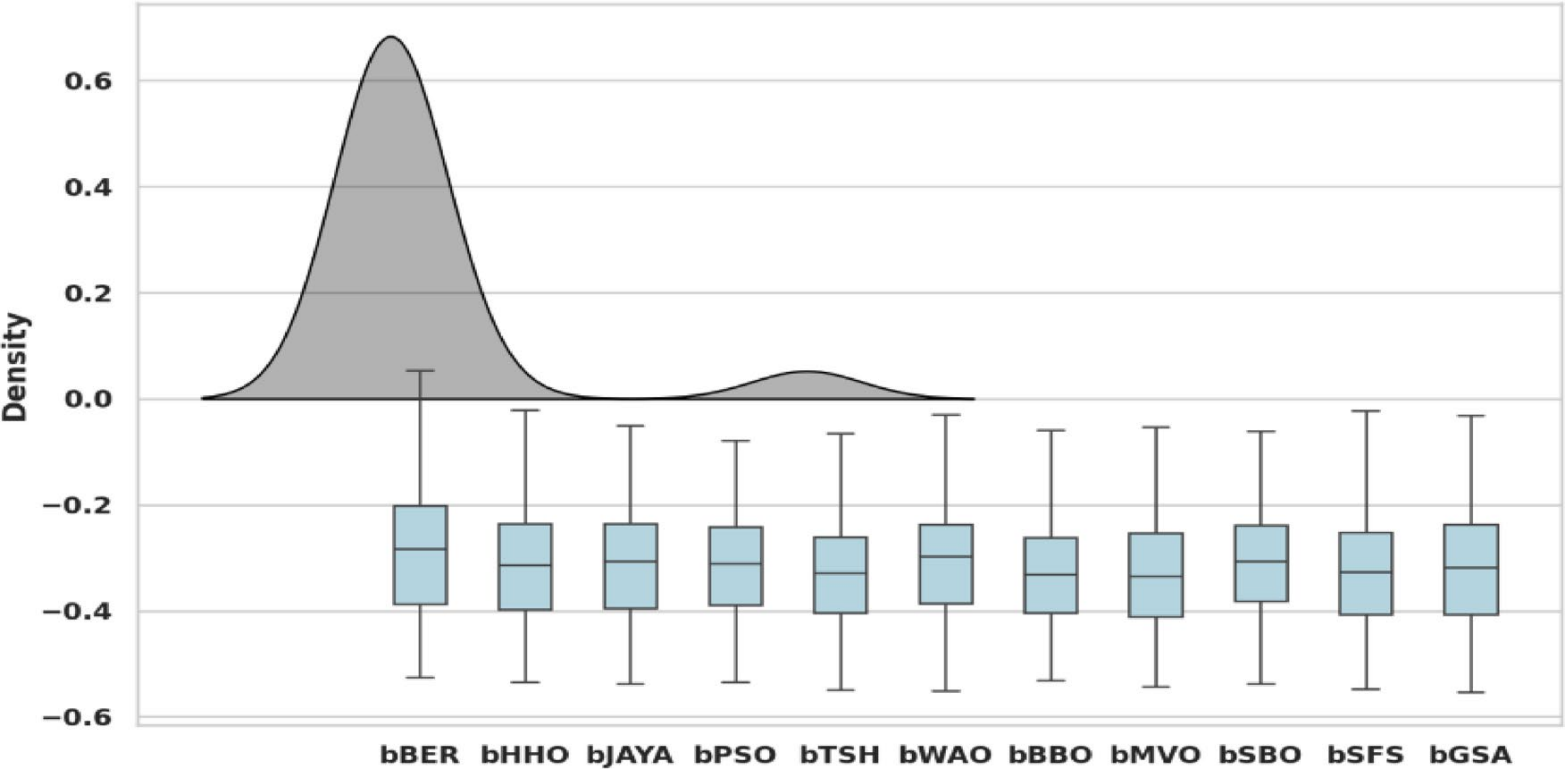
Boxplots with KDE overlay for proposed optimization algorithm with different other structures.

Figure 17 illustrates kernel density estimate (KDE) boxplots overlays for the proposed method versus many alternative techniques, with the bBER achieving the best results. Like a histogram, KDE graph is a way to visualize the distribution of occurrences in data. KDE uses a continuous probability density graph to describe the data in any of the dimensions. It shows the similarity scoring distributions that integrate confidence ratings and same/different choices. The chart indicates that, unlike other frameworks, bBER performs highest density. This figure strongly supports bBER’s superiority by combining statistical rigour (boxplots) and distributional nuance (KDE). Its narrow IQR and peaked KDE show unparalleled stability and optimality, giving it an excellent candidate for complicated optimization tasks.

### Classification results

Two fundamental ML models were tested and compared MLP and LSTM. The LSTM model outperforms MLP, achieving an accuracy of 0.958558559. Table 9 highlights the effectiveness of two ML approaches, assessed by the ACC., TRP, TNP, PPV, NPV, F1, and Time (s) metrics. The LSTM model outperforms others, achieving 0.9585%.

**Table 9:** Performance evaluation of classical ML models.

Models	Accuracy (ACC)	Sensitivity (TRP)	Specificity (TNP)	*P*-value (PPV)	*N*-value (NPV)	F1-Score	Time(s)
MLP	0.948198198	0.95215311	0.944680851	0.938679245	0.956896552	0.945368171	312.792
LSTM	**0.958558559**	0.95215311	0.962427746	0.938679245	0.970845481	0.945368171	**217.334**

The result of our proposed optimizer are in bold.

**Fig. 18:**
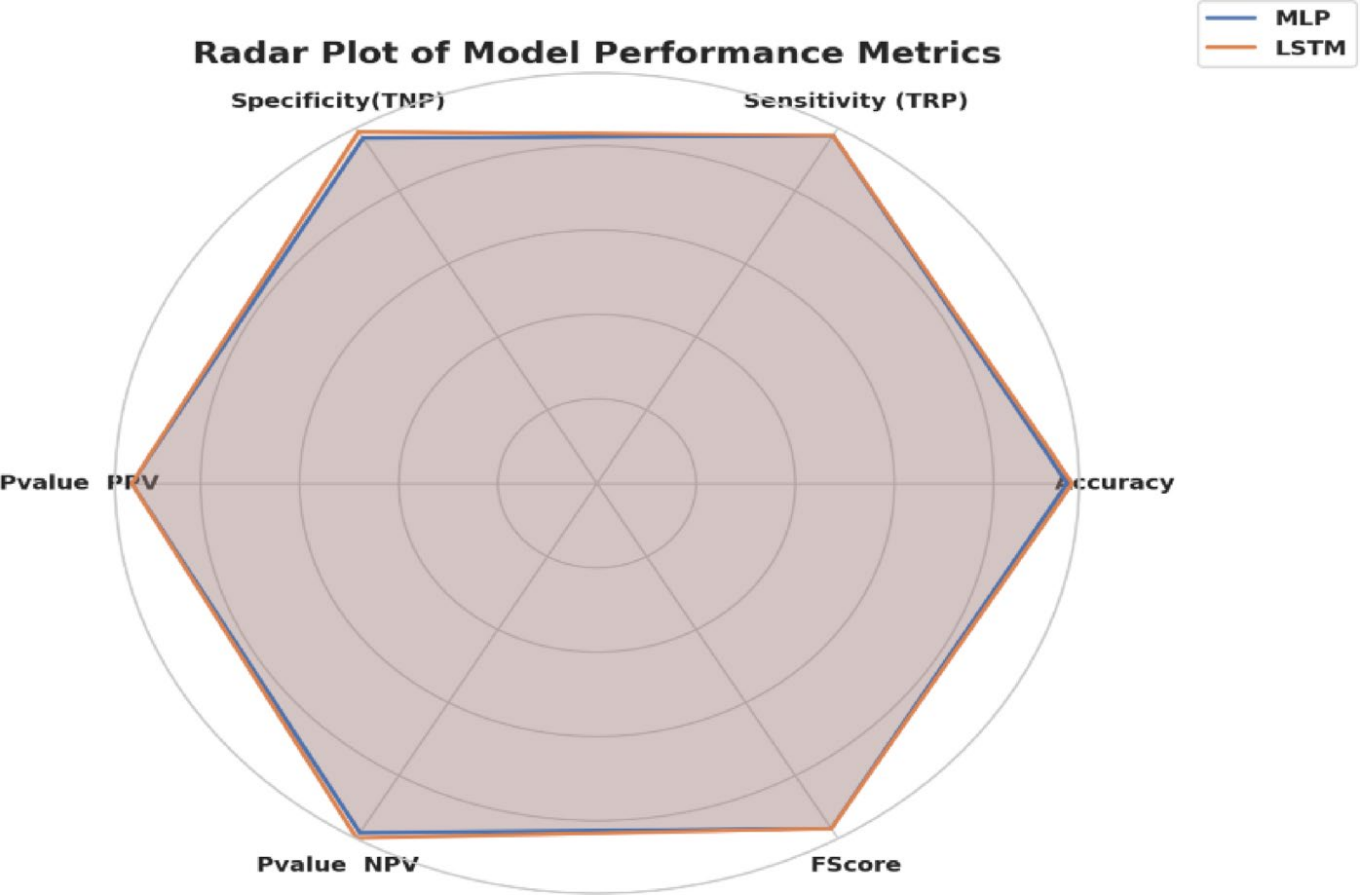
The radar plot of the bBER system’s capabilities matrix to the MLP and LSTM classifiers.

Figure 18 represents the Radar Plot of the bBER system’s efficiency metrics (ACC, TRP, TNP, PPV, NPV, F1, and Time (s)) to MLP and LSTM classifiers. It is concluded from the figure that LSTM was superior to MLP with achieving higher performance metrics.

**Fig. 19:**
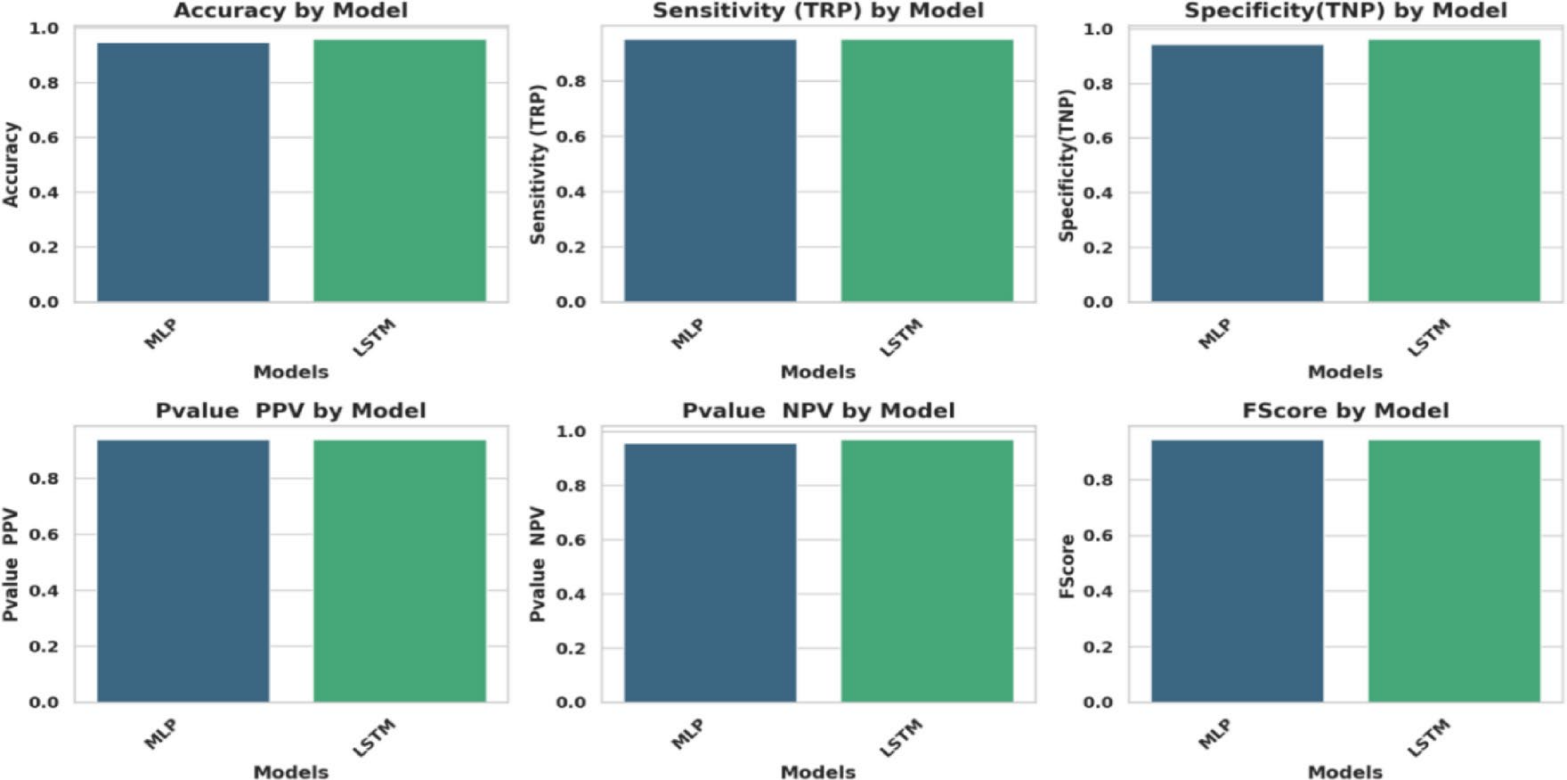
Evaluation of MLP and LSTM approaches using 6 metrics.

Figure 19 depicts an evaluation of the MLP and LSTM strategies using six metrics that show that the LSTM model outperforms MLP. Due to the achieved matrices by these two classifiers, LSTM outperforms MLP, as this chart indicates.

### Results of optimization model

Table 10 displays the results obtained by employing the recommended technique and other approaches to optimizing LSTM utilizing BER, HHO, PSO, JAYA, and SFS models. The statistical findings in Table 10 show that the BER+LSTM approach was superior to the other four optimizers utilizing LSTM models on benchmark functions. The BER+LSTM model surpassed previous cutting-edge classifier models developed using the LSTM technique, as indicated by its accuracy of 0.995260664. With a 0.987134503 accuracy, the HHO+LSTM-based technique produced the second-best classification results. It was followed by the PSO+LSTM-based technique, which scored 0.982826948; the JAYA+LSTM-based model, which scored 0.972555746; and the SFS+LSTM-based strategy, which generated the least accurate results of 0.962025316. Since the LSTM model performs better than simpler models, it is important to increase its accuracy; we intend to incorporate a new stage, including BER improvement. This technique could improve the system’s capacity to incorporate temporal variability in data, particularly for problematic stations such as TAM. The findings show that BER+LSTM continuously beats LSTM regarding RMSE for all stations and periods, with significant gains noted. To further verify generalization consistency, class-wise performance metrics were analyzed. The BER-optimized LSTM achieved 99.71% sensitivity and 99.31% specificity, yielding a balanced accuracy of 99.51%. The minimal discrepancy between class-wise metrics confirms that the model does not exhibit bias toward either Normal or Osteoarthritis classes and maintains stable performance across class distributions.

Table 11 shows the performance measures together with their 95% confidence ranges. The BER+LSTM model has extremely steady performance, as indicated by tight confidence intervals across all evaluation metrics. In comparison, models with lower overall performance had broader intervals, indicating more unpredictability. The minimal overlap between the confidence intervals of BER+LSTM and competing models supports the robustness and statistical relevance of the claimed performance gains.

**Table 10:** Classification outcomes for several LSTM-based optimization approaches.

Models	Accuracy	Sensitivity (TRP)	Specificity (TNP)	*P*-value (PPV)	*N*-value (NPV)	F-score	Time(S)
BER+LSTM	**0.995260664**	**0.99707887**	**0.993119266**	**0.994174757**	**0.996547756**	**0.995625**	**66.892**
HHO+LSTM	0.987134503	0.98798077	0.986332574	0.985611511	0.988584475	0.986795	82.817
PSO+LSTM	0.982826948	0.98325359	0.982300885	0.985611511	0.979411765	0.984431	93.216
JAYA+LSTM	0.972555746	0.97095436	0.973684211	0.962962963	0.979411765	0.966942	103.553
SFS+LSTM	0.962025316	0.95215311	0.968023256	0.947619048	0.970845481	0.949881	113.59

The result of our proposed optimizer are in bold.

**Table 11:** Performance metrics of all models on the test set (*N* = 845) with 95% confidence intervals, illustrating model stability and variability.

Model	ACC (95% CI)	TPR (95% CI)	TNR (95% CI)	PPV (95% CI)	NPV (95% CI)	F1 (95% CI)
BER+LSTM	99.53% (99.05–100%)	99.71% (99.35–100%)	99.31% (98.76–99.86%)	99.42% (98.91–99.92%)	99.65% (99.26–100%)	99.56% (99.09–100%)
HHO+LSTM	98.71% (97.97–99.46%)	98.80% (98.09–99.51%)	98.63% (97.86–99.41%)	98.56% (97.76–99.36%)	98.86% (98.18–99.54%)	98.68% (97.92–99.44%)
PSO+LSTM	98.28% (97.40–99.17%)	98.33% (97.47–99.18%)	98.23% (97.35–99.11%)	98.56% (97.76–99.36%)	97.94% (96.98–98.90%)	98.44% (97.61–99.28%)
JAYA+LSTM	97.26% (96.16–98.35%)	97.10% (95.96–98.23%)	97.37% (96.30–98.44%)	96.30% (95.04–97.55%)	97.94% (96.98–98.90%)	96.69% (95.49–97.89%)
SFS+LSTM	96.20% (94.91–97.49%)	95.22% (93.78–96.65%)	96.80% (95.64–97.97%)	94.76% (93.28–96.24%)	97.08% (95.95–98.22%)	94.99% (93.53–96.45%)

Table 12 shows statistical findings comparing the efficiency of the BER+LSTM algorithm to three different models (HHO, PSO, and JAYA) due to their best performance. The table shows that the BER+LSTM algorithm outperformed the other three models thanks to using two separate exploitation tactics in each cycle. The initial approach is to go in the direction of the majority ideal solution identified as of now, and the succeeding procedure is to seek superior options in close vicinity aggressively. Using these tactics, the BER+LSTM algorithm may optimize its utilization of the search space and get excellent results. Exploration and exploitation of the search space must be balanced to achieve effective exploitation. Furthermore, starting exploitation early in each cycle and progressively expanding the exploitation group’s size are vital.

**Table 12:** Comparison between the optimized BER+LSTM approach and the recent published approaches.

	BER+LSTM	HHO+LSTM	PSO+LSTM	JAYA+LSTM
Number of values	10	10	10	10
Minimum	0.9943	0.9801	0.9703	0.9653
25% Percentile	0.9953	0.9858	0.9828	0.972
Median	0.9953	0.9871	0.9828	0.9726
75% Percentile	0.9953	0.9871	0.9829	0.9726
Maximum	0.9963	0.9891	0.9883	0.9783
Mean	0.9953	0.9861	0.9822	0.9722
Std. Deviation	0.000471	0.002819	0.004508	0.003154

The statistical distinctions among the suggested approach and other comparable approaches are compared to confirm the approach’s performance utilizing ANOVA and Wilcoxon’s rank-sum tests. Table 13 shows the Wilcoxon Signed Rank test findings to the provided BER+LSTM approach and the comparative approaches used to ascertain whether statistically remarkable differences exist in the findings generated due to the approaches. A p-value which reached 0.002 implies statistically significant superiority. The findings show that the BER +LSTM framework is superior and of statistical significance.

**Table 13:** Wilcoxon signed-rank test for comparing the suggested (bBER) to alternative FS techniques.

	BER+LSTM	HHO+LSTM	PSO+LSTM	JAYA+LSTM	SFS+LSTM
Theoretical median	0	0	0	0	0
Actual median	0.9953	0.9871	0.9828	0.9726	0.962
Number of values	10	10	10	10	10
Wilcoxon signed rank test					
The sum of signed ranks (W)	55	55	55	55	55
The sum of positive ranks	55	55	55	55	55
The sum of hostile ranks	0	0	0	0	0
P value (two-tailed)	0.002	0.002	0.002	0.002	0.002
Is it exact or an estimate?	Exact	Exact	Exact	Exact	Exact
P-value summary	**	**	**	**	**
Significant (alpha = 0.05)?	Yes	Yes	Yes	Yes	Yes
How significant is the discrepancy?					
Discrepancy	0.9953	0.9871	0.9828	0.9726	0.962

Table 14 depicts the ANOVA test outcomes of the presented BER+LSTM framework versus the comparison approaches. Both the ANOVA and Wilcoxon signed-rank tests indicate the BER+LSTM approach’s superior efficacy, statistically verifying its improved performance in KOA diagnosis. These studies not only verify the stability of the suggested approach but also highlight its significant difference compared to other approaches, reinforcing its reliability for accurate detection of KOA.

**Table 14:** The ANOVA outcomes to the presented bBER approach with LSTM model for KOA categorization.

	SS	DF	MS	F (DF_*n*_, DF_d_)	*P* value
Treatment (between columns)	0.006464	4	0.001616	F (4, 45) = 120.2	*P* < 0.0001
Residual (within columns)	0.000605	45	1.34E-05		
Total	0.007069	49			

**Fig. 20:**
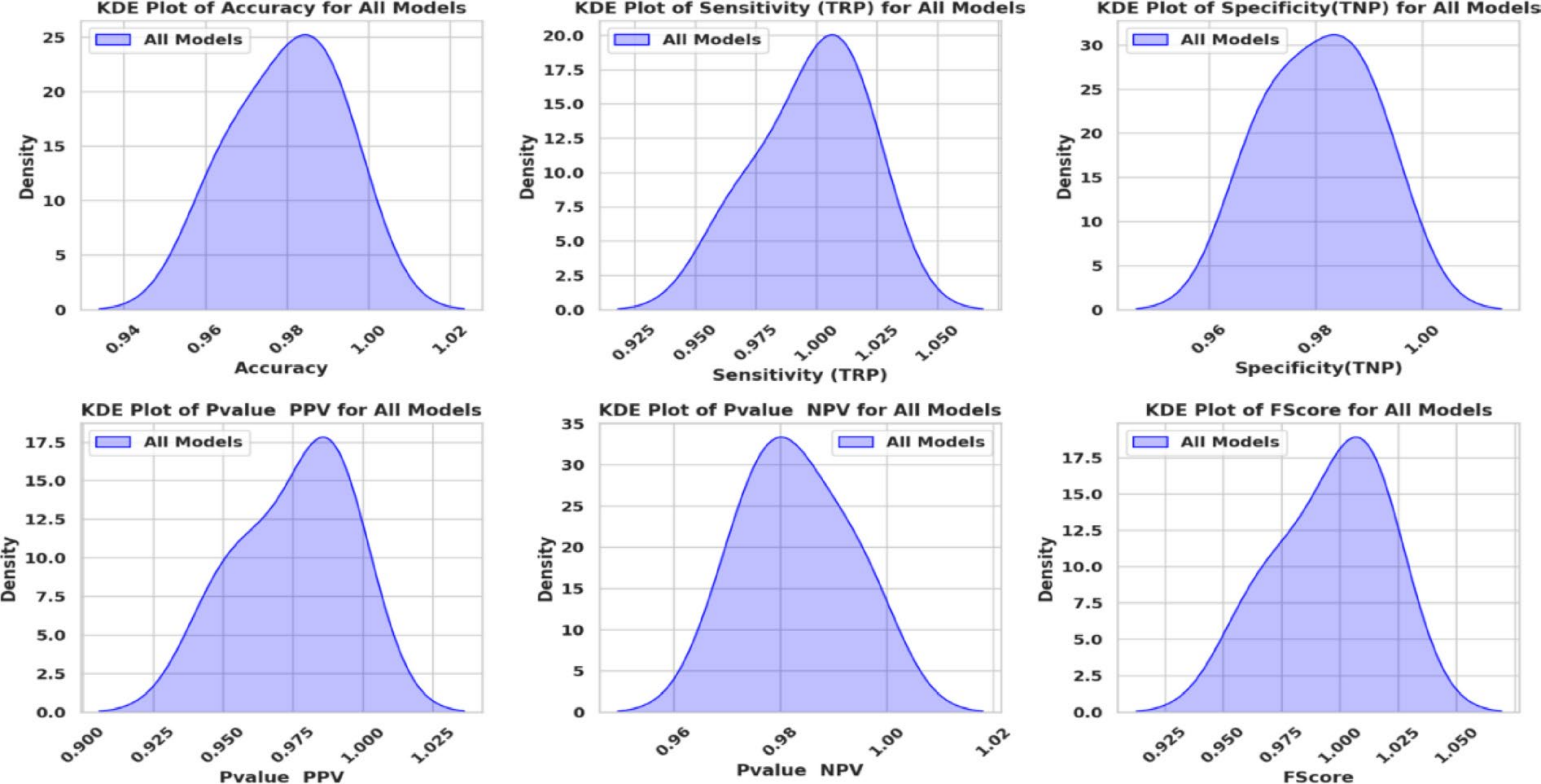
Kernel density estimation plots for model metrics are employed to optimize algorithms.

Figure 20 shows KDE charts for model metrics (ACC, TRP, TNP, PPV, NPV, F1, and Time(s)) in optimization models. KDE creates a graphic that’s easier to understand and less crowded than a histogram, mainly if exhibiting numerous distributes. It is a crucial illustration, exhibiting the framework’s effectiveness, robustness, and longevity. Researchers can compare the accuracy efficiency of the suggested methodology to alternative approaches and identify any underlying trends in the distribution using the KDE plot. In the context of KOA categorization, this visualization provides a thorough means of assessing the relative efficacy and overall prediction capacity of each approach.

**Fig. 21:**
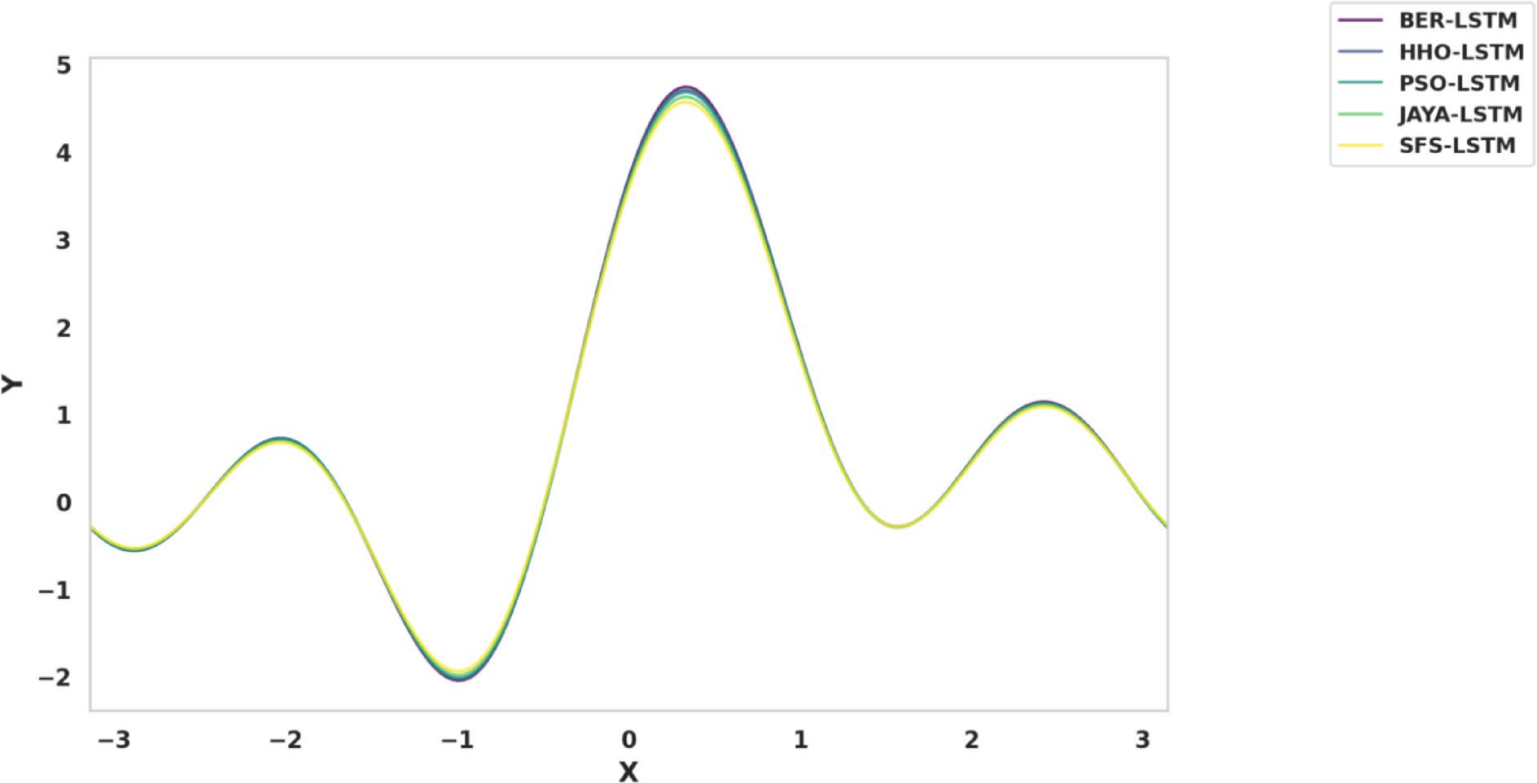
Andrews curves for model performance metrics on the presented BER+LSTM framework and other comparable systems.

Figure 21 illustrates the Andrews curves for model performance measures for the proposed BER+LSTM and other comparable approaches. As can be concluded from the Figure, the suggested approach performs better than other alternative approaches. The Andrews curves in Fig. 21 show that the BERT+LSTM architecture is consistently dominant over the evaluation spectrum. Its harmonious curve shape shows balanced strengths in FS (BER) and sequence modelling (LSTM), whereas competitors have fragmented skills.

**Fig. 22:**
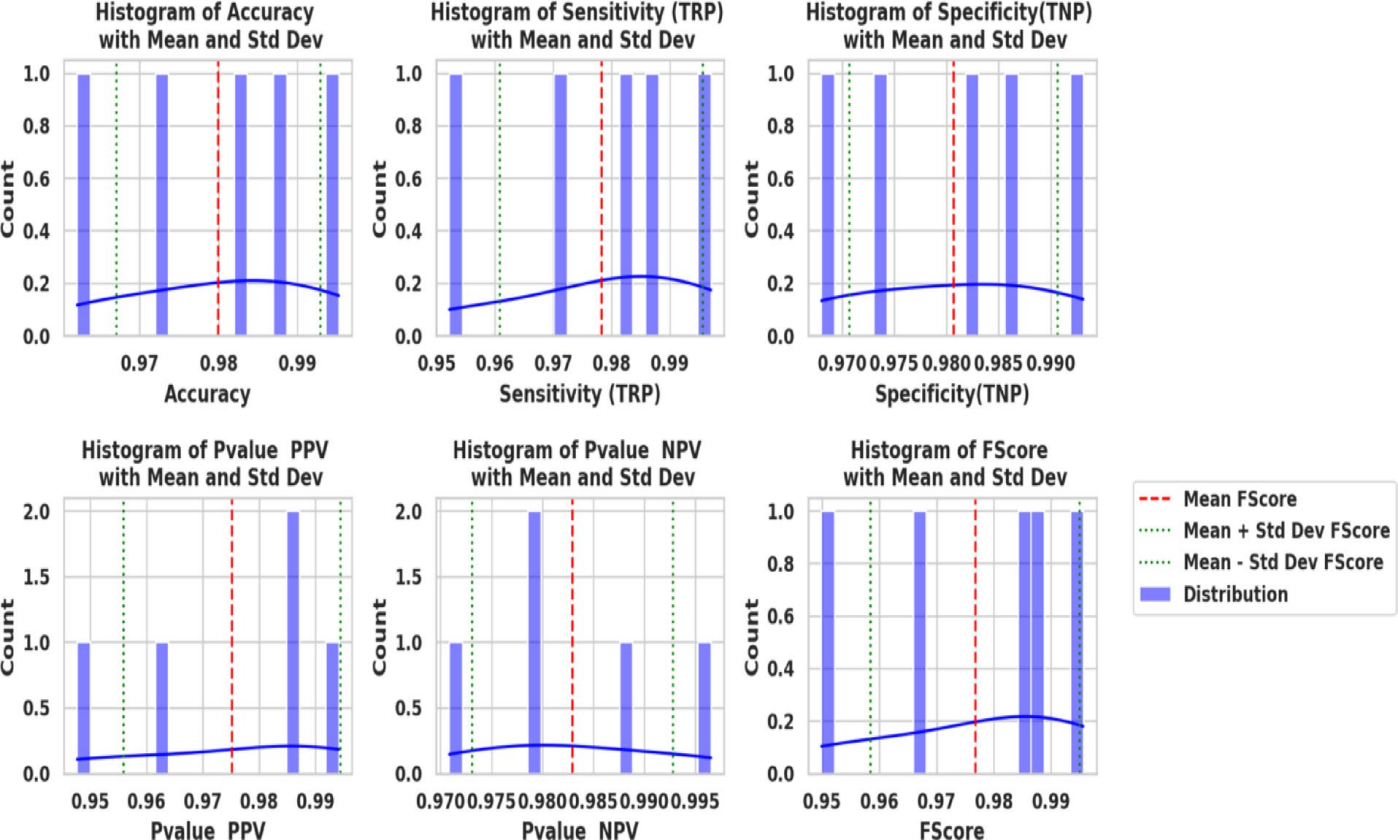
Histograms of the six metrics among models, showing mean and standard deviation.

Figure 22 depicts the histograms of the six metrics across models with mean and standard deviation and distribution parameters, where the dashed red lines show the mean F score, and the dashed green lines show the mean and standard deviation F score. It is frequently deployed to display the key characteristics of the data distributed conveniently.

**Fig. 23:**
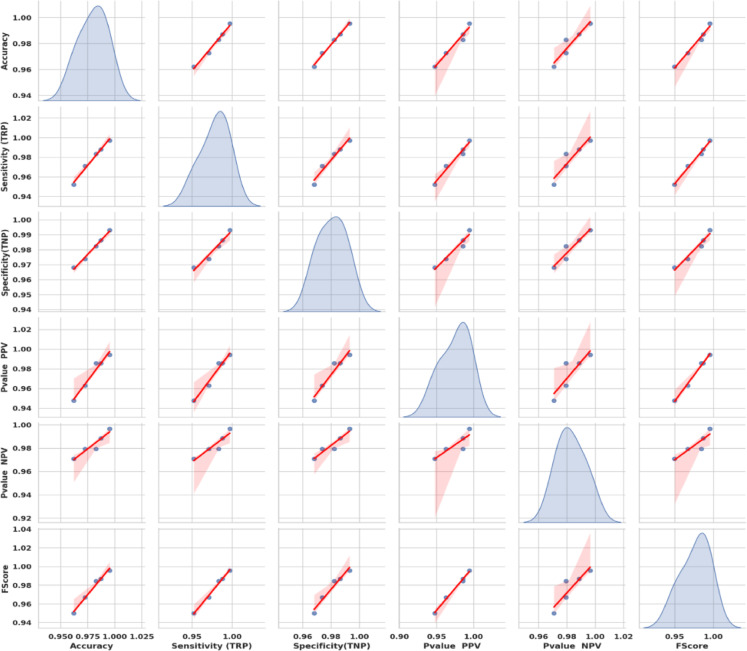
Metric pair plots with regression lines to the suggested BER+LSTM framework and optimization models.

Figure 23 depicts a pair plot of measurements with regression lines, which provides a detailed visualization of the relationship between various efficiency metrics for the BER+LSTM technique and alternative techniques. The graphic allows us to explore the relationships between numerous metrics, such as ACC, TRP, TNP, and F1 score, which are given in pairs. The relevant regression line shows the direction and intensity of the association for each pair of metrics, which is displayed as a scatter plot. These regression lines are very effective for spotting trends or patterns in data and emphasizing potential links between various performance indicators. Analyzing the above pair plot, researchers may get important insights into the interactions between measurements across several strategies. Furthermore, by contrasting the regression lines to the BER+LSTM process with those of the other computations, a relative performance evaluation may be performed, identifying both areas of strength and opportunities for enhancement in the organization of KOA assignments.

**Fig. 24:**
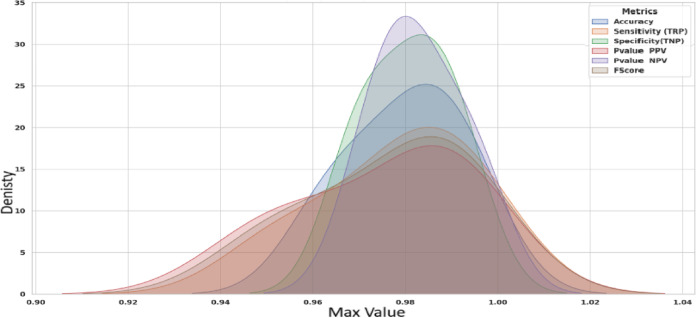
KDE plot of accuracy of the suggested BER+LSTM framework findings and other algorithms compared to multiple metrics.

Figure 24 depicts a KDE plot of the accuracy of reference model results when compared across many criteria, illustrating the distribution of the BER+LSTM accuracy scores technique and a variety of reference techniques. This graphic provides a thorough kernel density estimate, allowing for a more complex understanding of accuracy score variability and dispersion among various techniques. Each method is depicted as a probability density curve, where higher peaks signify regions of greater concentration and denser clustering of accuracy scores. Researchers may contrast the accuracy of the BER+LSTM methodology to alternative procedures and find any underlying trends in the distribution using the KDE plot. In the context of KOA categorization, this graphic provides a comprehensive tool for evaluating each algorithm’s relative effectiveness and total prediction capabilities categorization. The KDE figure shows the overall advantage of the BERT-LSTM framework, which achieves balanced sensitivity/specificity, greater accuracy, and statistically significant results. Its dependability for mission-critical applications is confirmed by its tight, right-shifted distributions across all measures.

**Fig. 25:**
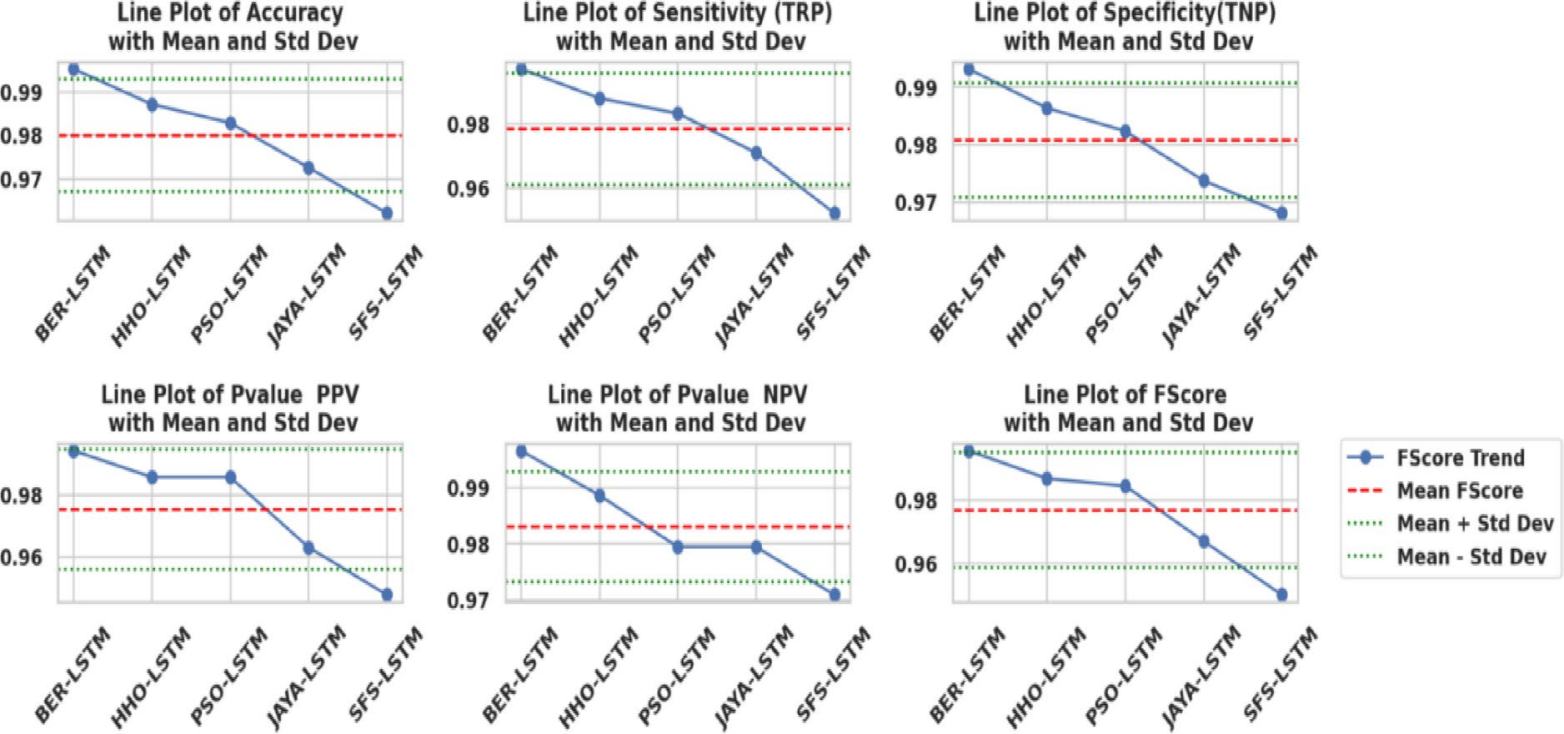
Line plot with mean and standard deviation for metrics across different optimization models.

Figure 25 depicts a line plot with mean and standard deviations for metrics from several optimization models, including BER+LSTM, HHO+LSTM, PSO+LSTM, JAYA+LSTM, and SFS+LSTM. The end-to-end superiority of BERT-LSTM is illustrated in this figure by its increased accuracy, balanced clinical metrics, and stable statistical significance. Its dependability for practical implementation is highlighted by the small error bands on all lines.

**Fig. 26:**
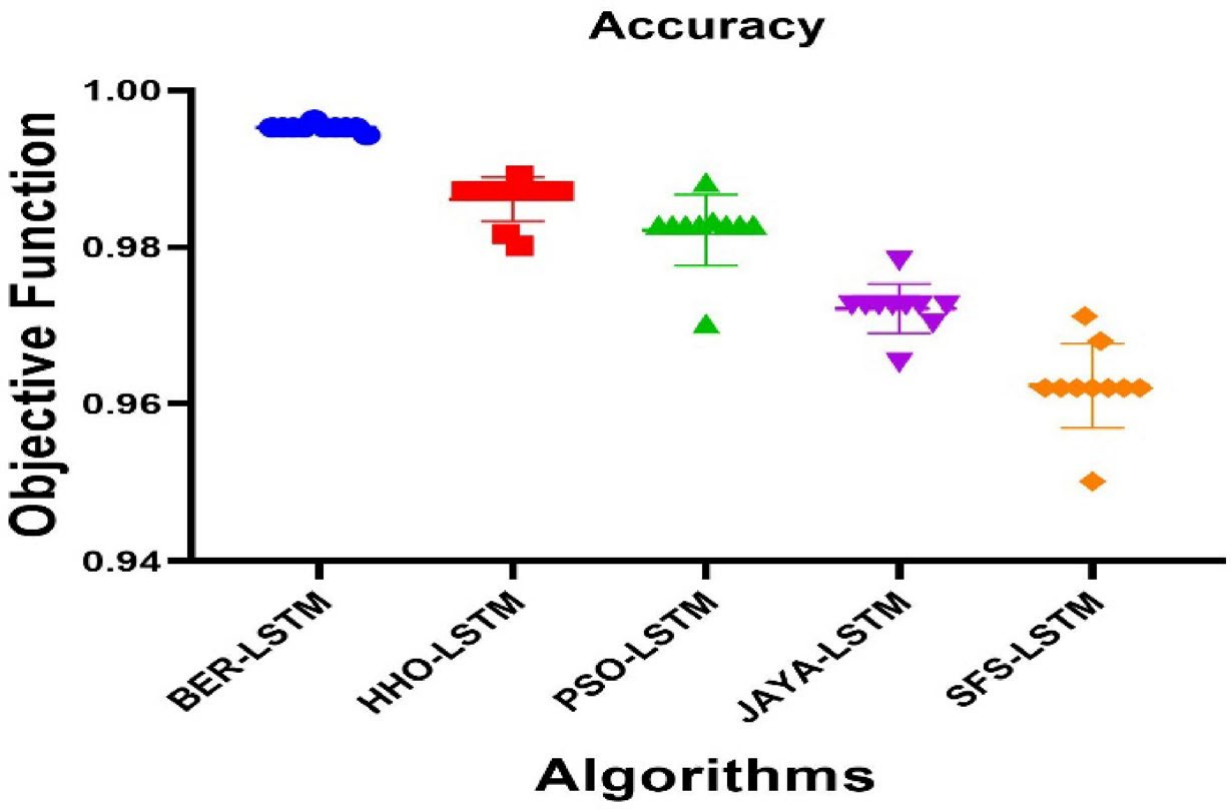
Accuracy plot for suggested optimization algorithm compared to other algorithms utilising the LSTM classifier.

Figure 26 depicts an accuracy plot for the suggested BER+LSTM method in contrast to existing optimization algorithms used on the LSTM procedure, concentrating on the objective function. This research evaluates the BER+LSTM method’s efficiency in optimizing the objective function for KOA categorization by contrasting it to other techniques. The findings contribute to a better knowledge of the proportional efficacy of different strategies, setting a standard for their practical implementation. This visual depiction enables academics and practitioners to identify all technique’s unique strengths and limits, allowing them to choose the best strategy for enhancing KOA classification models.

**Fig. 27:**
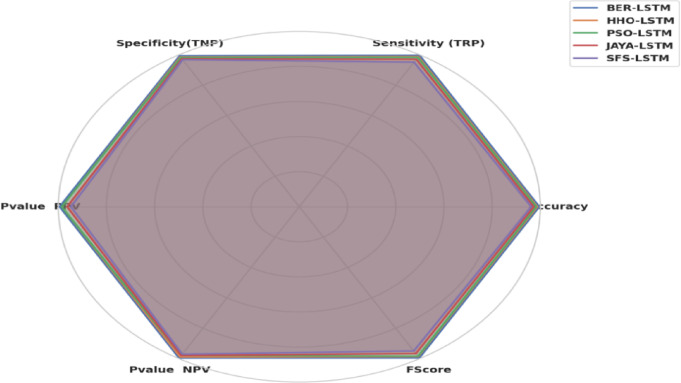
Radar plot of model efficiency matrix with other models.

Figure 27 depicts a radar plot of the Model Performance Matrix with various approaches: BER+LSTM, HHO+LSTM, PSO+LSTM, JAYA+LSTM, and SFS+LSTM. This graphic indicates that the recommended strategy outperformed the other feasible strategies. The figure indicates that BERT+LSTM as particularly balanced and high-performing hybrid due to its spacious, symmetrical radar profile. Its superior sensitivity and specificity make it suited for applications with significant misclassification costs.

**Fig. 28:**
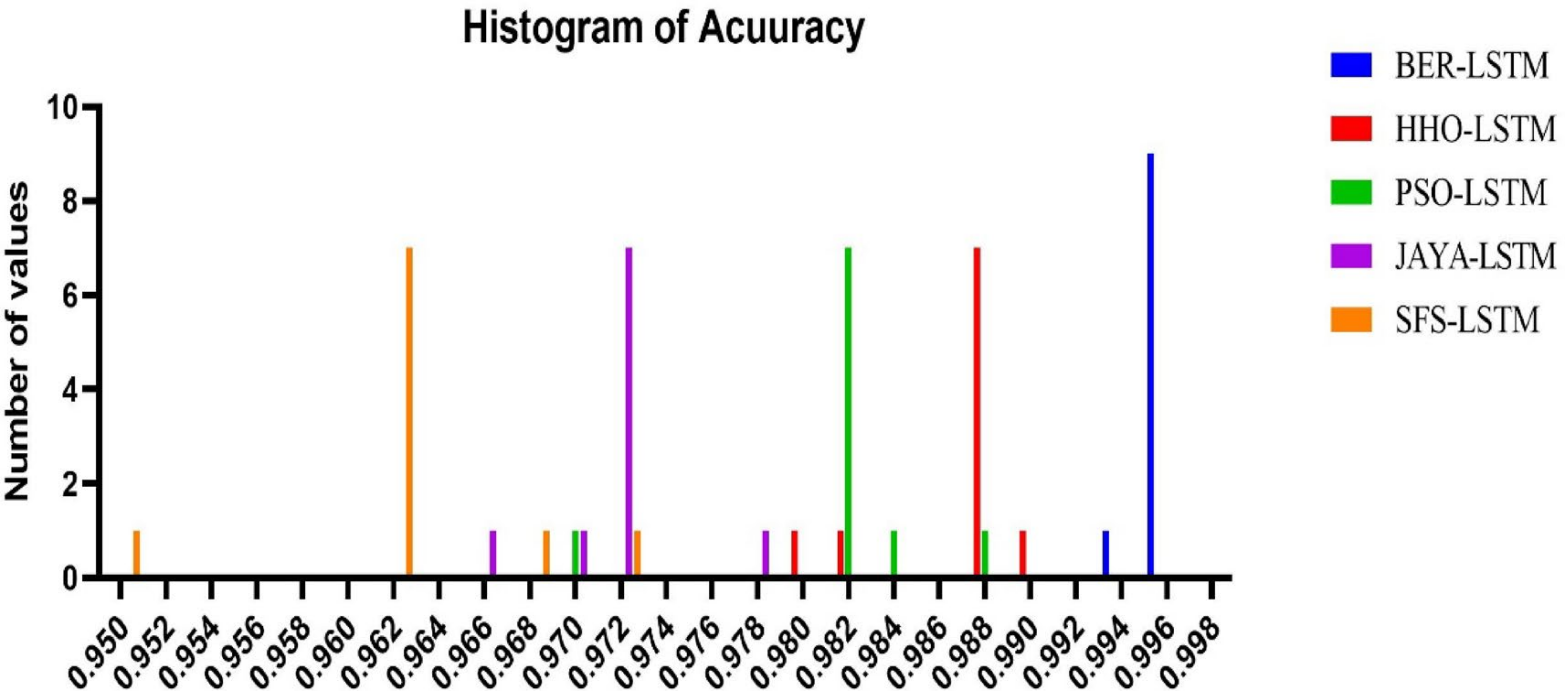
Histogram of accuracy scores obtained by the proposed BER+LSTM algorithm and alternative combinations of optimized approaches with LSTM-based approach.

Figure 28 depicts a histogram of the accuracy of the presented BER+LSTM strategy compared to alternative optimization strategies for LSTM procedures. These histograms clearly show the variety and distribution of accuracy ratings over several approaches, providing helpful information about their effectiveness. Researchers can comprehend the data distribution better by examining these illustrations, which enables them to spot trends, outliers, and potential areas for methodological improvement. The histograms in Fig. 28 allow for relevant conclusions about the performance of distinct optimization techniques when combined with LSTM methods for KOA categorization problems.

**Fig. 29:**
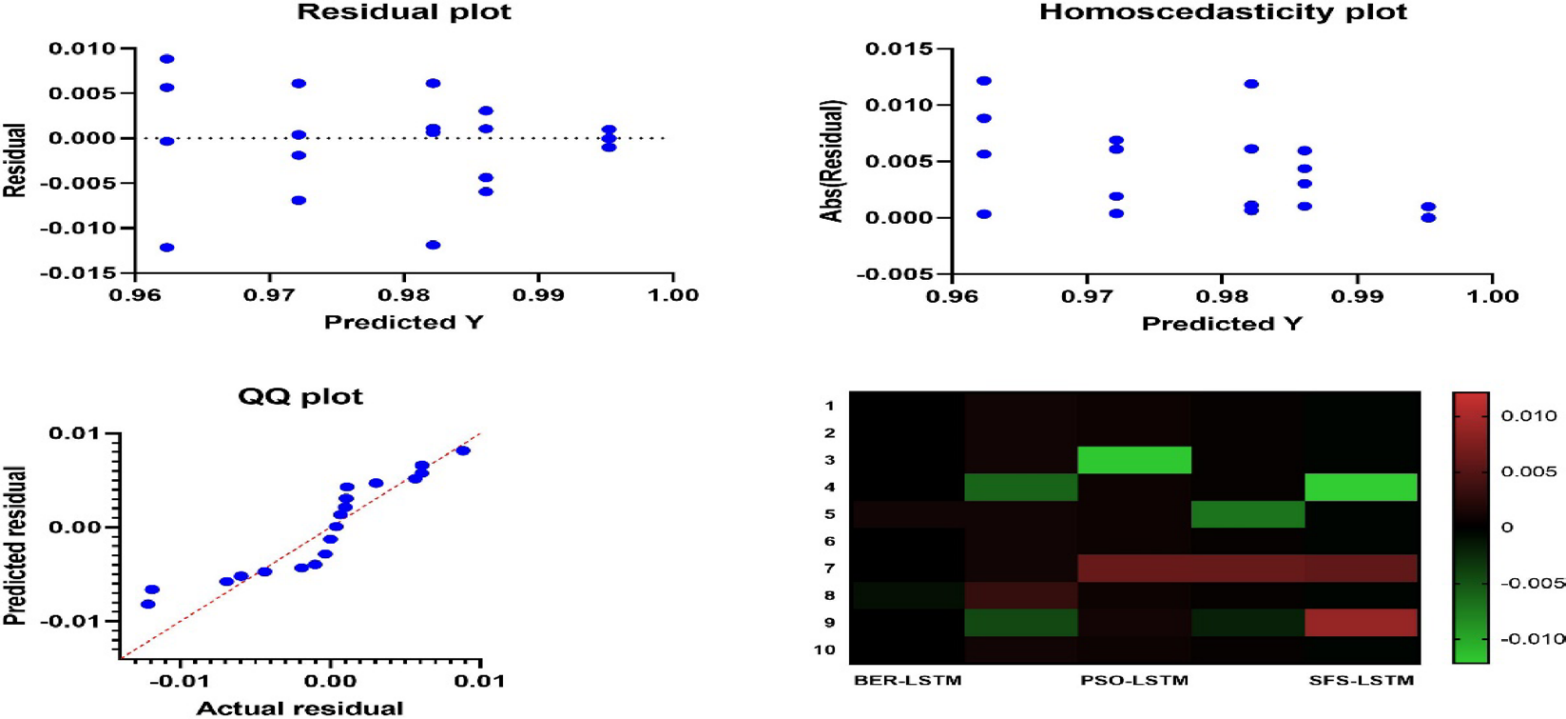
Analysis charts show the findings for BER+LSTM, PSO+LSTM, and SFS+LSTM.

**Fig. A1:**
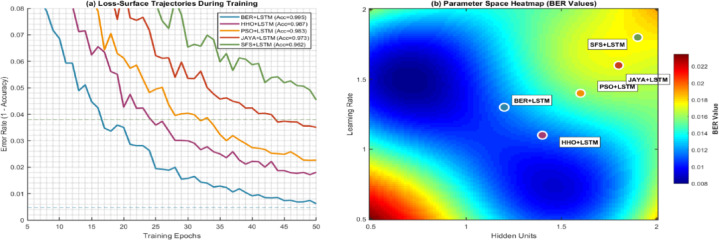
Convergence behavior through two complementary views. (**a**) loss-surface trajectories. (**b**) parameter-space heatmap.

**Fig. A2:**
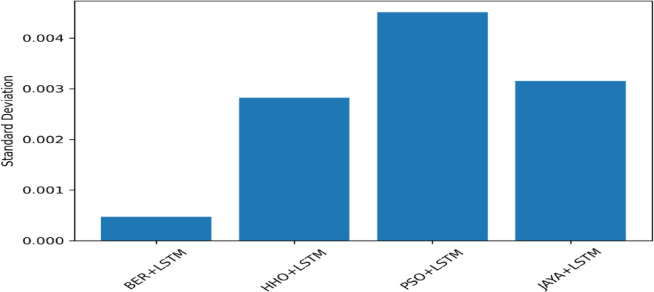
Stability comparison based on standard deviation across 10 independent runs.

**Fig. A3:**
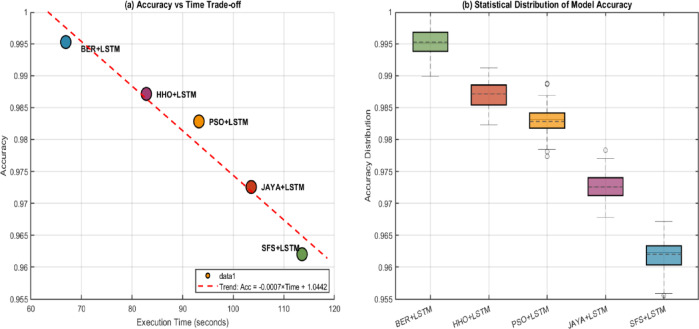
Statistical robustness. (**a**) Accuracy vs Time trade-off. (**b**) Box plots.

Figure 29 shows this scenario’s heat map, QQ plot, residual plot, and heteroscedasticity plot. The dots in the QQ plot closely match the line, indicating a linear connection between the actual and anticipated residuals. This demonstrates the efficiency of the suggested strategy for categorizing KOAs. The analysis graphs in Figure demonstrate the usefulness of the suggested technique in settling the optimization difficulties addressed in this work. Detailed convergence analysis, multi-metric comparison, and statistical validation are provided in Figures A1, A2, A3 in the Appendix.

**Fig. 30:**
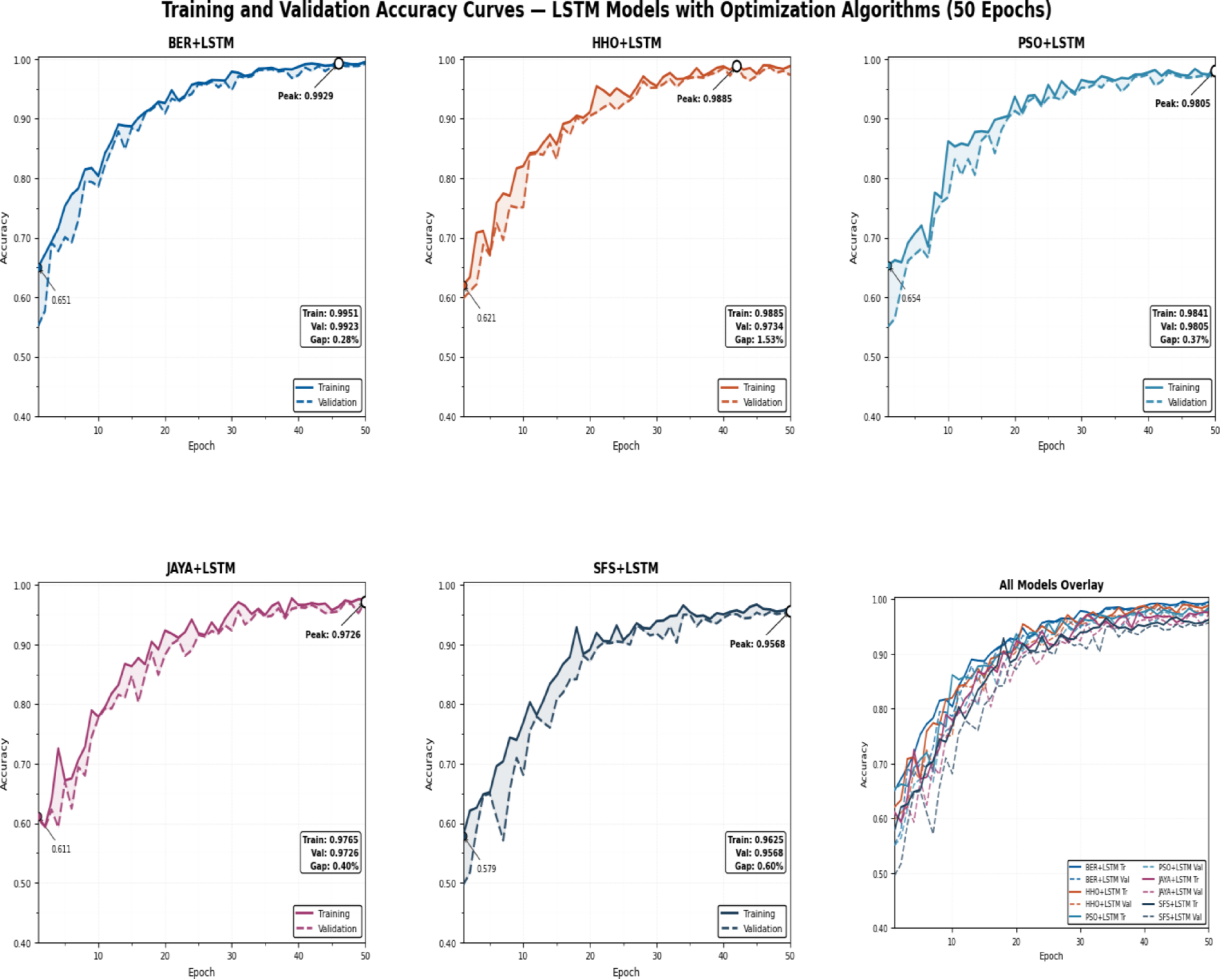
Training and validation accuracy curves over 50 epochs for LSTM models optimized utilizing different metaheuristic algorithms (BER, HHO, PSO, JAYA, and SFS).

Figure 30. depicts the training and validation accuracy curves over 50 epochs for LSTM models optimized using different metaheuristic algorithms (BER, HHO, PSO, JAYA, and SFS). The curves illustrate the convergence behaviour and learning stability of each model. The small gaps between training and validation accuracy indicate strong generalization capability and limited overfitting. The BER–LSTM model achieves the highest validation accuracy and the most stable convergence pattern. The small training–validation gaps observed across epochs indicate stable convergence and strong generalization performance of the proposed framework.

### Computational complexity and resource analysis

**Fig. 31:**
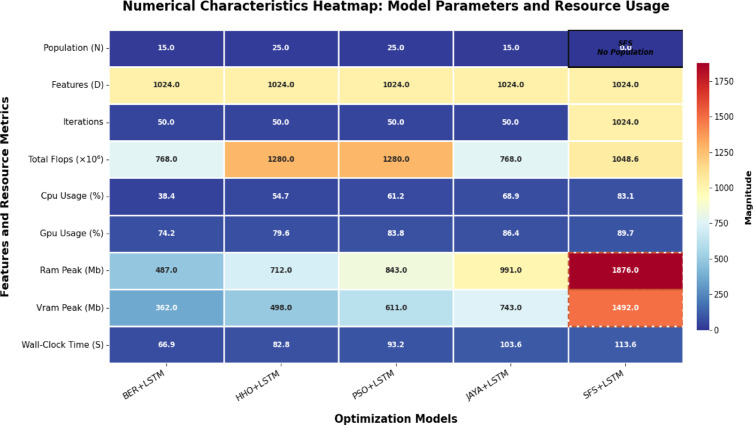
Comparative analysis of computational resource utilization across the evaluated optimization models. The figure reports the total floating-point operations (FLOPs), CPU and GPU utilization, peak RAM and VRAM consumption, and the overall wall-clock execution time.

Theoretical and empirical analysis were used to investigate the computational needs of the evaluated optimisation techniques. Table 15 summarises the key parameters influencing computational complexity, such as population size (n), number of features (d), and number of iterations (iter). Therefore, the overall time complexity of the studied optimisation algorithms is O(n⋅d⋅iter). In addition to the theoretical investigations, an empirical assessment of computing resource utilisation was conducted. As shown in Fig. 31, numerous parameters were investigated, including total floating-point operations (FLOPs), CPU and GPU utilisation, peak RAM and VRAM consumption, and overall execution time. The results show that the BER+LSTM framework strikes an appropriate compromise between prediction performance and computational economy. Methods such as HHO, PSO, JAYA, and, in particular, SFS, demand significantly more computer resources. The higher cost of the SFS-based approach is mostly owing to the fact that the number of iterations grows with the number of features, considerably increasing the overall computing workload.

**Table 15:** Theoretical computational complexity and actual execution time for BER+LSTM and optimizer-based LSTM models.

Model	Population (*n*)	Features (d)	Iterations	Complexity	Total FLOPs (×10^6^)	CPU Usage (%)	GPU Usage (%)	RAM Peak (MB)	VRAM Peak (MB)	Wall-clock Time (s)
BER+LSTM	15	1024	50	O(15·1024·50)	768	38.4	74.2	487	362	66.892
HHO+LSTM	25	1024	50	O(25·1024·50)	1280	54.7	79.6	712	498	82.817
PSO+LSTM	25	1024	50	O(25·1024·50)	1280	61.2	83.8	843	611	93.216
JAYA+LSTM	15	1024	50	O(15·1024·50)	768	68.9	86.4	991	743	103.553
SFS+LSTM		1024	1024	O(d²) O(1024²)	1048.6	83.1	89.7	1876	1492	113.59

**Fig. 32:**
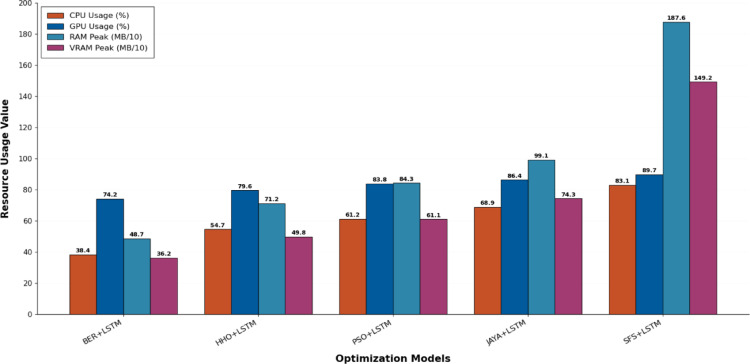
Computational resource consumption across LSTM- based optimization models (CPU/GPU: Percentage, RAM/VRAM: MB/10).

**Fig. 33:**
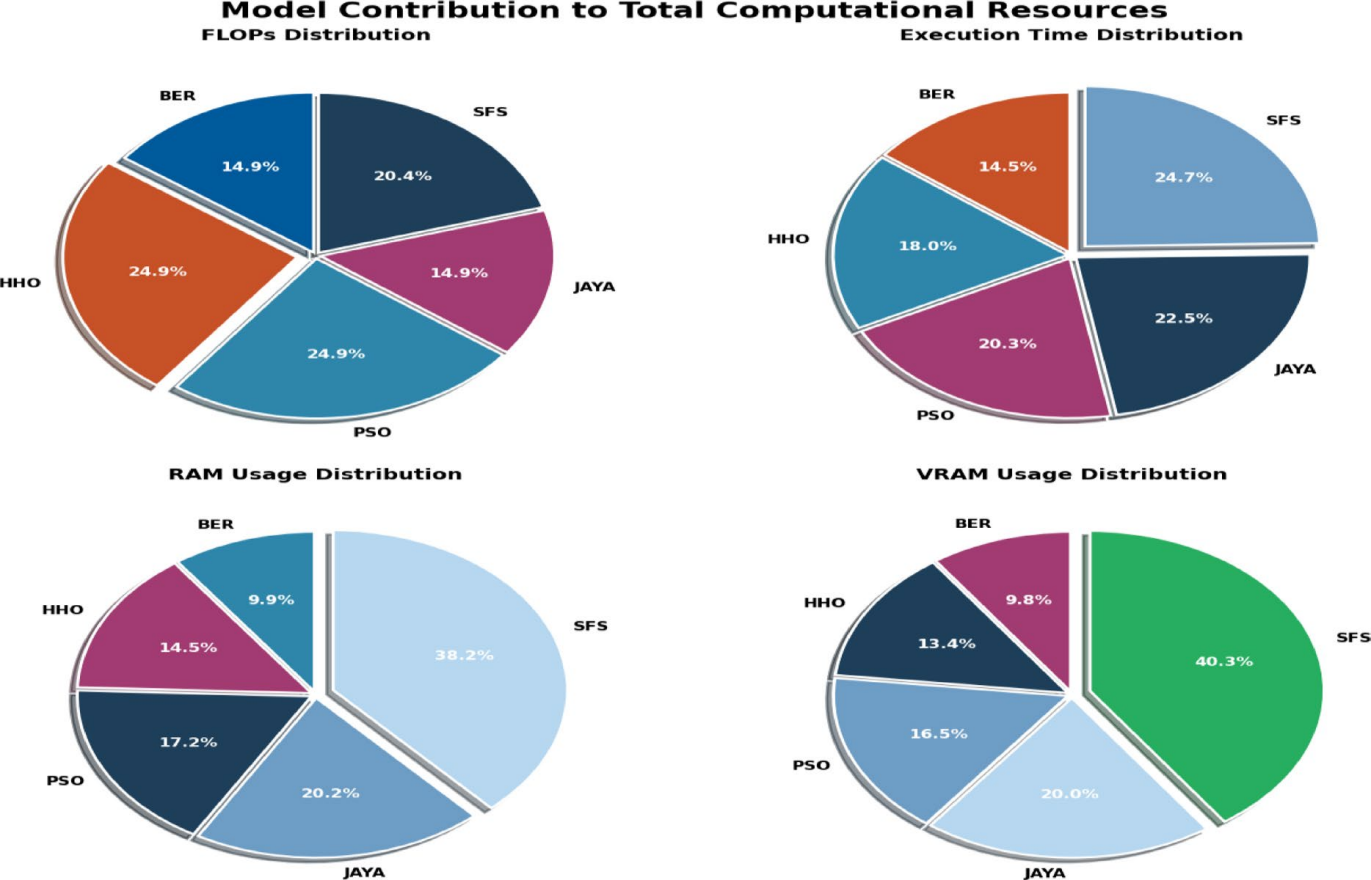
Comparative contribution of optimization methods to total computational resource utilization (FLOPs, Execution time, RAM, and VRAM).

Figure 32 shows the computational resource consumption of different LSTM-based optimization models. CPU and GPU usage are represented as percentages, and RAM/VRAM usage in MB. The proposed model achieves the lowest resource consumption across all metrics, indicating superior efficiency compared to other models. The figure highlights that the proposed model requires less CPU/GPU utilization and minimal memory resources, making it more suitable for deployment on hardware with limited computational capacity. Figure 33 presents the contribution of each evaluated optimization method to the overall computational resource consumption. The analysis includes four key metrics: floating-point operations (FLOPs), execution time, RAM usage, and VRAM usage. As shown in the figure, the SFS-based approach exhibits the highest computational demand across memory-related metrics, accounting for approximately 38.2% of RAM usage and 40.3% of VRAM consumption. In contrast, the BER-based approach demonstrates significantly lower resource requirements, contributing only about 9.9% of RAM usage and 9.8% of VRAM usage. Similar trends are observed in terms of FLOPs and execution time, where BER maintains a relatively lower computational footprint compared with other optimization methods such as HHO, PSO, and JAYA. These findings further highlight the efficiency of the BER+LSTM framework in achieving competitive performance while maintaining lower computational resource consumption.

### Robustness analysis

#### Robustness to class imbalance

The dataset contains 1,589 (Normal) and 2,246 (Osteoarthritis) knee X-ray images, demonstrating a considerable class imbalance. To ensure that model performance did not favour the dominant class, evaluation went beyond overall accuracy to include class-sensitive indicators. The balanced accuracy was calculated using Eq. 9 as shown below.

9 $$~Balanced~Accuracy = \frac{{Sensitivity + Specificity}}{2}$$

Tables 16 and 17 show the calculations of the balanced accuracy for the baseline LSTM model and proposed BER+LSTM model, which closely matches its overall accuracy 0.995099 (for optimization model), indicating balanced performance across Normal and OA classes.

**Table 16:** Calculations of the balanced accuracy for the LSTM and MLP models.

Models	Balanced Accuracy
MLP	0.948417
LSTM	0.95729

**Table 17:** Calculations of the balanced accuracy for the proposed BER+LSTM model and other optimization approach.

Model	Balanced Accuracy
BER+LSTM	0.995099
HHO+LSTM	0.987157
PSO+LSTM	0.982777
JAYA+LSTM	0.972319
SFS+LSTM	0.960088

The high degree of agreement between overall accuracy 99.52% and balanced accuracy 99.51% demonstrates that classification performance was not influenced by class distribution bias. Furthermore, the remarkable alignment of Sensitivity 0.9971 and Specificity 0.9931 indicates symmetric discriminative competence across both Normal and OA classes. In addition, high Positive Predictive Value 0.9942 and Negative Predictive Value 0.9965 (as indicated from Table 10) indicate stable predictive reliability across both classes, further supporting balanced model behavior.

**Fig. 34:**
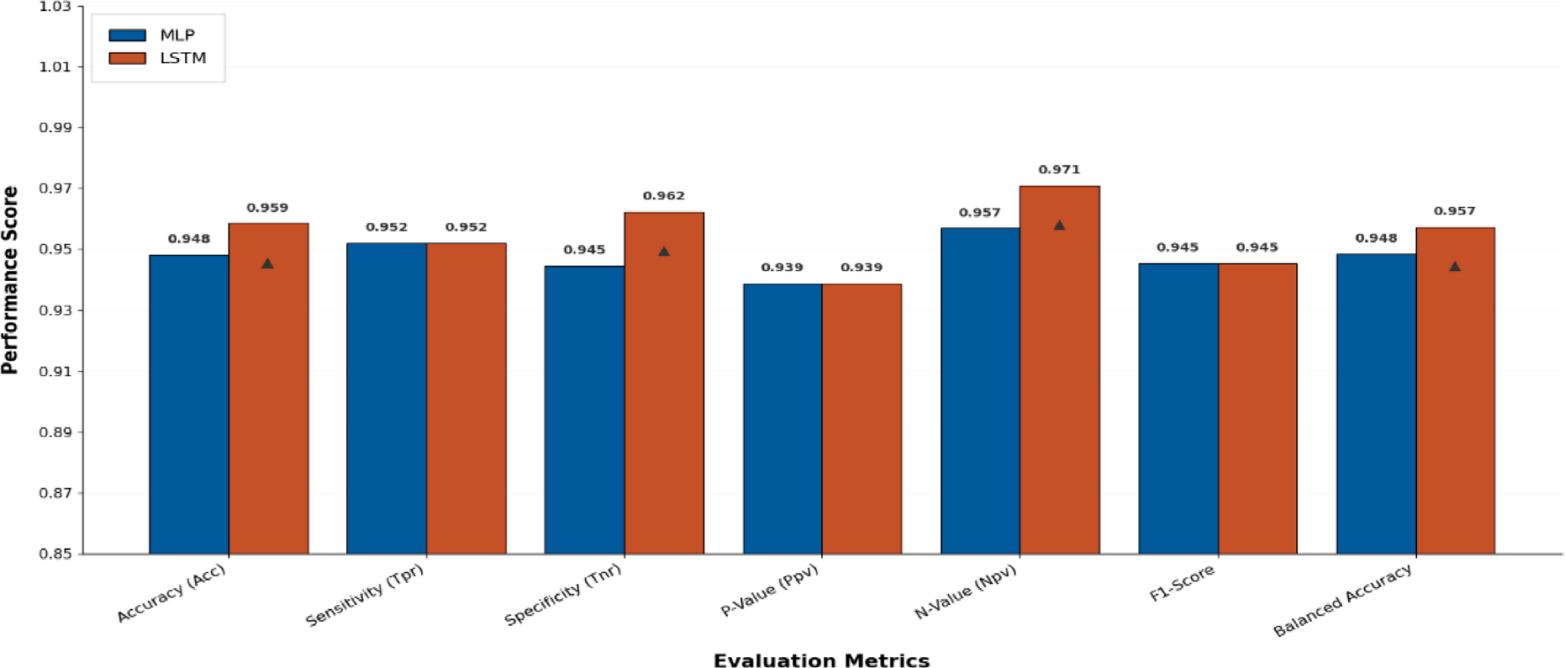
Comparative analysis MLP and LSTM model performance classification metrics.

Figure 34. depicts a comparative performance analysis of MLP and LSTM models across multiple classification metrics. The LSTM model consistently outperforms the MLP across most metrics, particularly in Specificity, NPV, and Balanced Accuracy, indicating superior and more balanced classification performance.

**Fig. 35:**
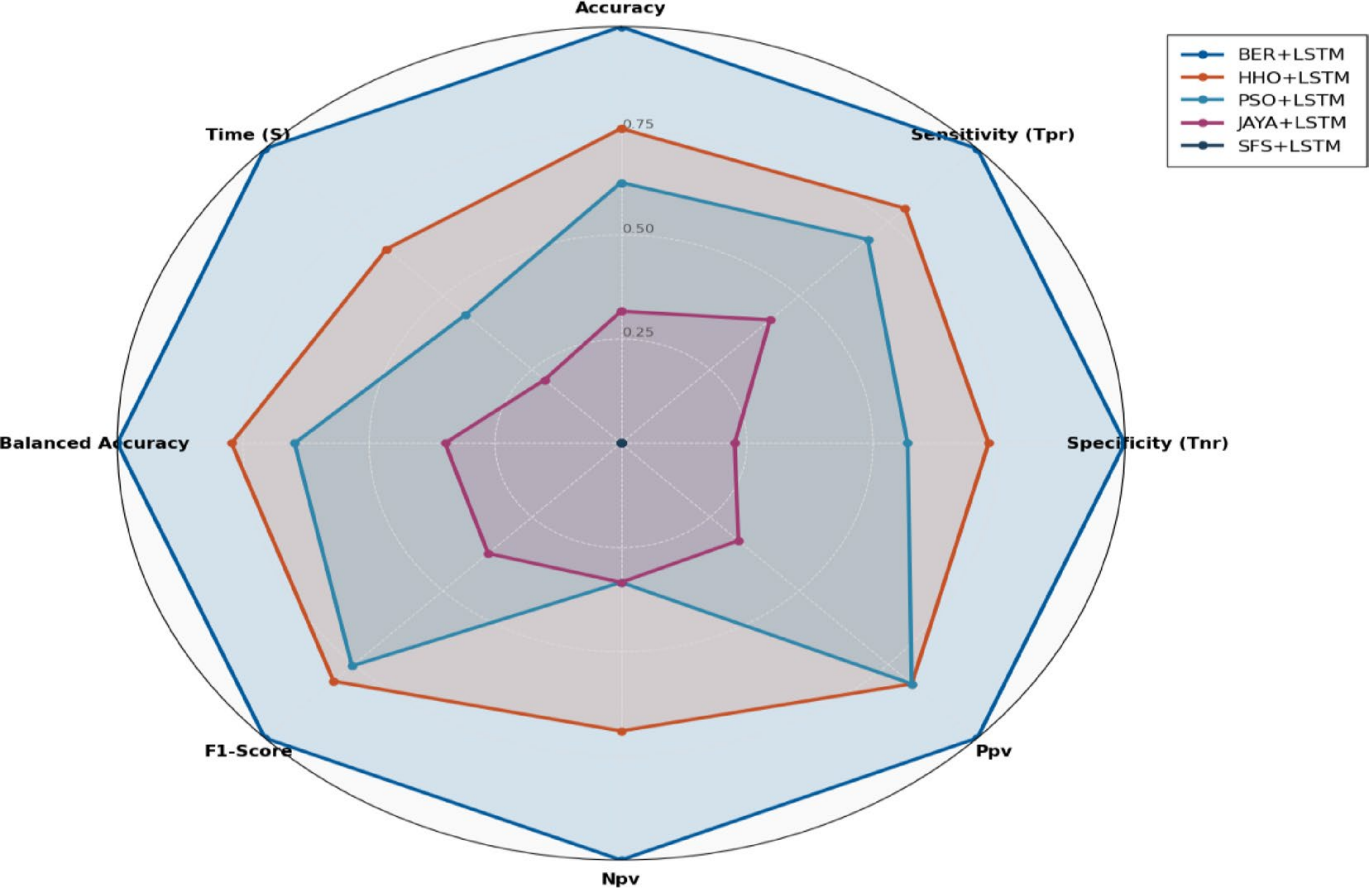
The radar chart of the performance of different LSTM-based models across multiple normalized metrics, including Accuracy, Sensitivity (TPR), Specificity (TNR), PPV, NPV, F1-score, Balanced accuracy, and Time.

Figure 35. Depicts the radar chart of the performance of different LSTM-based models across multiple metrics, including Accuracy, Sensitivity (TPR), Specificity (TNR), PPV, NPV, F1-score, Balanced Accuracy, and Time. Balanced Accuracy, which averages Sensitivity and Specificity, reflects the model’s ability to correctly classify both positive and negative cases. The BER+LSTM model achieves the highest Balanced Accuracy (~ 0.995), indicating superior and balanced classification performance compared to other models. HHO+LSTM and PSO+LSTM follow closely, while JAYA+LSTM and SFS+LSTM show lower values, reflecting less balanced performance.

**Fig. 36:**
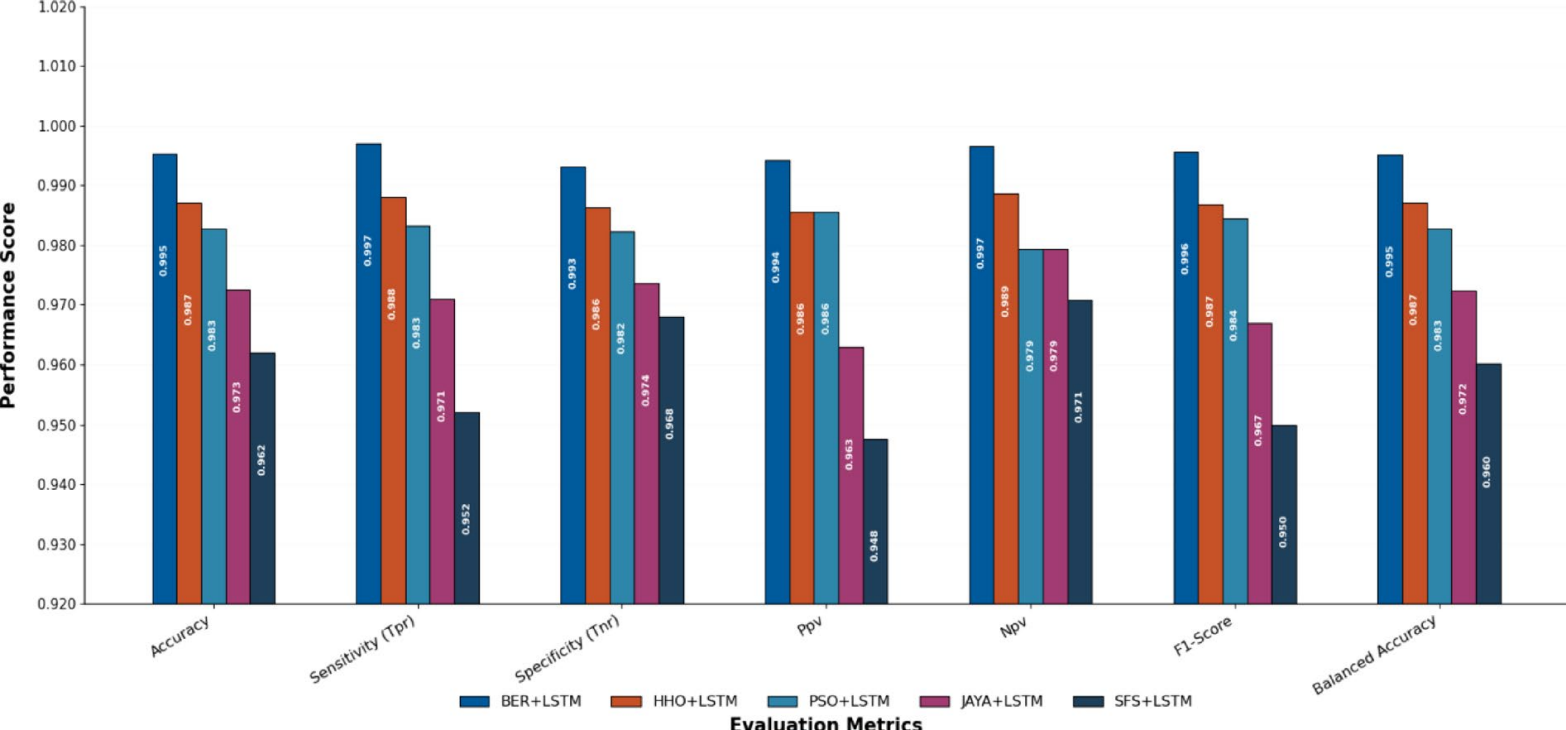
Comprehensive model performance comparison classification metrics analysis across all models.

Figure 36. Shows the comprehensive model performance comparison classification metrics analysis across all models. As indicated from the figure that, the proposed BER+LSTM achieves the highest scores across all metrics, demonstrating superior classification performance and robustness compared to alternative models.

#### Robustness to X-ray acquisition variability

Although the dataset originates from a single public source, variability in X-ray imaging may arise from differences in anatomical positioning, minor orientation shifts, and exposure conditions.

To mitigate potential acquisition-related variability, several preprocessing and training strategies were employed:

- Intensity scaling and normalization to standardize pixel distributions.
- Removal of null entries to ensure clean and consistent inputs.
- Data augmentation during training, including horizontal flipping and controlled rotational transformations.

Horizontal flipping simulates left–right anatomical orientation differences, while rotational augmentation introduces invariance to minor positioning misalignments during image acquisition. These strategies enhance spatial invariance and reduce model sensitivity to positional variability.

Furthermore, the low standard deviation observed across repeated experimental runs deflects strong experimental stability and reproducibility.

#### Statistical validation of model stability

To quantitatively assess performance differences among optimization strategies, statistical analyses were conducted.

A one-way ANOVA (see Table 14) demonstrated a statistically significant difference between models. This indicates that observed performance improvements are not attributable to random variation. The low within-group variance (MS = 1.34 × 10⁻⁵) further supports experimental consistency.

Following pairwise tests using the Wilcoxon signed-rank test (see Table 13), the BER+LSTM configuration was found to be statistically superior to other optimization algorithms (HHO, PSO, JAYA, and SFS). Collectively, these statistical findings give compelling evidence that the suggested optimization approach produces consistent, reproducible, and statistically significant performance improvements.

### Comparison with literature

As we explained previously, the accuracy and efficacy of the suggested approach were evaluated by comparing it to other models; it outperformed all of them, demonstrating its effectiveness. These findings showed how well the BER+LSTM approach established a nonlinear relationship between operational conditions and process responsiveness. Furthermore, it offered Improved statistical performance metrics for the KOA prediction and categorization compared to the other models that were considered. When compared to the Fire-Fly Algorithm (FFA), ACO Optimizer, and GAO Optimizer, the suggested method produces superior outcomes and validates its efficacy for KOA diagnosis and evaluation. The Table 18 compares the knee joint detection techniques with the traditional systems in situations of atypical KOA Amjad Rehman et al., Isra Malik et al., S. Kavitha et al., B. Subha et al., and Teemu A. T. et al. The comparative table indicates evident that the hybrid bBER model has produced superior metrics than the methods used in prior studies. Table 18 shows that, in compared to all previous approaches, the proposed method’s detection accuracy is noticeably higher and the time required to run the model is brief. This is due to the bBER optimization technique was employed to optimize the weights in the LSTM.

**Table 18:** Comparisons of the suggested approach in this study to comparable publications.

Reference	Dataset/size	Methods	Precision	Time (s)
^34^	3615 images	2D-CNN CRK model CNN ML classifier: RF and KNN	99%	13.07
^41^	OAI	Ensemble TL-ACO Alex-Net custom Isr-Net k-means clustering ACO optimizer	89.89%	-
^42^	Kaggle	Texture- and color-based FE CNN Firefly approach optimization	CNN model is improved by 2.5%	-
^43^	Captured from the humans /2283	GAO Optimizer Novel Dual CNN (DCNN)	98.77%	0.0004
^44^	OAI/1213	RF Self-coded MATLAB graphical user interface	65.9%	-
Proposed Method	OAI/3835	Alex-Net, VGG19-Net, and Google-Net bBER optimizer LSTM and MLP classifier	**99.526%**	**66.892**

Significant values are in bold.

## Conclusion

This work introduces the BER method, which attempts to increase the precision of KOA classification. The BER optimizer’s binary format aims to select the optimal features to raise classification accuracy. A set of assessment criteria was utilized to evaluate the suggested strategies. The ANOVA and Wilcoxon signed-rank tests were utilized in the statistical analysis to compare the suggested algorithm’s relevance and efficacy to 10 different algorithms (HHO, JAYA, SBO, GSA, SFS, MVO, BBO, WOA, PSO, and TSH). In addition, many illustrations are developed to show the resilience and effectiveness of the suggested technique. Visually representations of the results were also created to test the suggested algorithm’s strength and usefulness. The practical and statistical results generally show that the proposed technique outperforms other competing optimization algorithms for KOA classification, with a 0.995260664 accuracy. This study illustrates the effectiveness of the BER approach in FS and hyperparameter adjustment.

Despite the outstanding efficacy of the proposed framework, certain limitations should be highlighted. First, the model is based on a relatively small dataset, and variations in radiography acquisition settings may limit its generalization capacity. Second, the lack of additional clinical or demographic metadata limits the system to unimodal image-based inference. Third, while sequential reformulation of CNN feature vectors for LSTM processing is theoretically justifiable, it may fail to fully retain the intrinsic spatial topology of convolutional representations. Future research will involve evaluating the suggested technique against larger and more diversified multi-center datasets in order to properly assess its resilience and scalability. Furthermore, new optimization techniques and advanced segmentation algorithms might be investigated to improve feature refinement and region-of-interest extraction. Further research into new radiographic symptoms of osteoarthritis and their relationship to therapeutic therapies may also improve the clinical usefulness of the proposed paradigm and establish its definite strengths and limits.

## Data Availability

The data that support the findings of this study are openly available at [https://www.kaggle.com/datasets/farjanakabirsamanta/osteoarthritis-prediction].

## Appendix

The comparative performance analysis of the optimized LSTM models is illustrated in Figures A1, A2, A3. As shown in Figure A1, the BER-optimized LSTM achieves the highest classification accuracy (99.52%), outperforming HHO, PSO, JAYA, and SFS. Stability analysis presented in Figure A2 reveals that BER exhibits the smallest standard deviation across multiple independent runs, confirming its robustness and resistance to random initialization effects. Furthermore, the convergence behavior illustrated in Figure A3 indicates that BER converges faster and more smoothly toward the optimal solution compared to other metaheuristic optimizers. The reduced oscillatory behavior suggests an effective balance between exploration and exploitation mechanisms.
